# Minimal-moderate variation of human oral virome and microbiome in IgA deficiency

**DOI:** 10.1038/s41598-021-94507-8

**Published:** 2021-07-21

**Authors:** Maria José de la Cruz Peña, Luis Ignacio Gonzalez-Granado, Inmaculada Garcia-Heredia, Lucia Maestre Carballa, Manuel Martinez-Garcia

**Affiliations:** 1 Department of Physiology, Genetics, and Microbiology, University of Alicante, Alicante, Spain; 2 Primary Immunodeficiencies Unit, Pediatrics, Hospital 12 Octubre, Instituto de Investigación Hospital 12 octubre (imas12), Madrid, Spain; 3 School of Medicine, Complutense University, Madrid, Spain

**Keywords:** Immunology, Microbiology, Diseases, Medical research

## Abstract

Immunoglobulin A (IgA) is the dominant antibody found in our mucosal secretions and has long been recognized to play an important role in protecting our epithelium from pathogens. Recently, IgA has been shown to be involved in gut homeostatic regulation by ‘recognizing’ and shaping our commensal microbes. Paradoxically, yet selective IgA-deficiency is often described as asymptomatic and there is a paucity of studies only focused on the mice and human gut microbiome context fully ignoring other niches of our body and our commensal viruses. Here, we used as a model the human oral cavity and employed a holistic view and studied the impact of IgA deficiency and also common variable IgA and IgM immunodeficiencies (CVID), on both the human virome and microbiome. Unexpectedly, metagenomic and experimental data in human IgA deficiency and CVID indicate minimal-moderate changes in microbiome and virome composition compared to healthy control group and point out to a rather functional, resilient oral commensal viruses and microbes. However, a significant depletion (two fold) of bacterial cells (p-value < 0.01) and viruses was observed in IgA-deficiency. Our results demonstrate that, within the limits of our cohort, IgA role is not critical for maintaining a rather functional salivary microbiome and suggest that IgA is not a major influence on the composition of abundant commensal microbes.

## Introduction

Secretory immunoglobulin A (IgA) is the dominant antibody in mucosal secretions and is produced by plasma cells in the lamina propria and poly Ig receptor-mediated secretion by epithelial cells overlying mucosal surfaces^1^. It is well known that IgA plays an essential role in defense against microbial pathogens^2^. Both, T cell-dependent and independent IgA responses can be generated^3^. Secretory IgA has the ability to recognize multiple antigenic epitopes on the surface of pathogenic viruses and bacteria and consequently prevents the adhesion and penetration the epithelium^1^. However, the specificity and recognition of these epitopes and more interestingly the immune adaptative responses and mechanisms to distinguish between commensal and pathogenic microbes is not fully understood and remain mostly enigmatic^4,5^. We strongly recommend for a more complete picture of IgA long and recent-standing biology, the excellent recent review by Pabst and Slack^3^. We now know that microbiome also plays a paramount role in the induction and education of the host immune system by a complex ‘cross-talk’ to maintain a stable mutualistic relationship^6^. A remarkable effort to that end was the discovery of the natural polyreactivity features of IgA to coat several gut commensal bacteria^7^ (i.e. cross-species reactivity) that seem to point to an existing endogenous mechanism driving homeostatic production of polyreactive IgA with innate specificity to our microbiota^7^. It has been proposed that IgA responses can be co-opted by the microbiome to engender robust host-microbial symbiosis and commensal gut microbes use this antibody for mucosal colonization^8^. Thus, these studies and others (see a recent comprehensive review by Pabst and Slack^3^) have broadened our view and point that IgA seems to play a role shaping commensal microbiome and maintaining an adequate equilibrium on host-microbiome symbiosis^7–10^. Paradoxically, IgA-deficiency is often asymptomatic or mild symptoms in humans, which intuitively challenge that pivotal role as a fundamental mechanism to recognize our commensal microbiome. The effect of IgA-deficiency on human microbiome composition has been addressed in mice-models^11^ and in the human gut, fully ignoring the effect in other niches of our body, such as the oral mucosal and cavity. In the human gut, on one side, it has been proposed that IgA-deficient humans exhibit a gut microbiota dysbiosis^12^, while other experimental data support that IgA deficiency does not lead to massive, major perturbations in the gut microbiome^11^. Furthermore, all these recent microbiome studies have fully ignored our extremely abundant commensal viruses. Thus, it is uncertain whether IgA plays an irreplaceable role for recognizing our commensal microbes and viruses. Paraphrasing a recent review by Pabst and Slack^3^, ‘IgA is a divisive molecule and this Odyssey seems particular confusing’.

Here, to address these questions, we analyzed and discussed the impact of IgA deficiency on the oral microbiome employing a more holistic view considering in our microbiome study the viral community structure^13,14^ along with changes in microbial abundance (viruses and bacteria) between control and IgA deficiency groups.

## Results

### 16S rRNA gene amplicon sequencing

**Figure 1:**
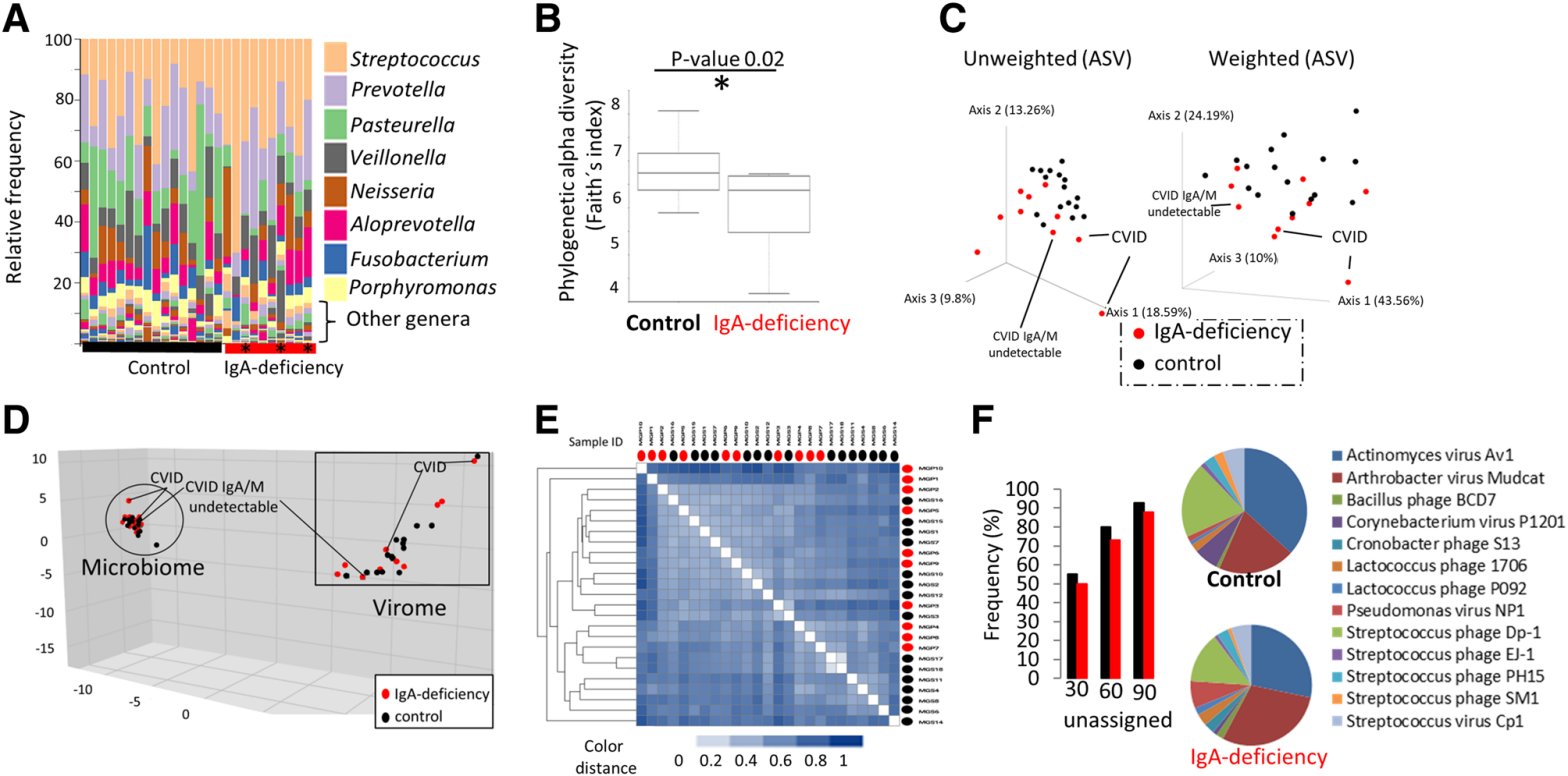
Diversity, composition and metabolic functionality of human oral microbiome and virome from control and IgA deficiency. (**A**) Taxonomic analysis at the genus level based on 16S rRNA gene Illumina amplicon sequencing. Relative abundance (%) of the most abundant genera (at least > 1% of abundance) is displayed. For convenience, these other rare taxa are not depicted in panel enumerating species. Star denotes CVID patients. (**B**) Comparison of phylogenetic alpha richness diversity by Faith’s index between groups. Other alpha diversity indices used in Qiime program were not conclusive (p-value > 0.05). ANCOM test implemented in Qiime2 did not found statistical differences for common abundant bacterial commensalists. (**C**) PCoA representing Unweighted and Weighted Unifrac distance for controls and IgA-deficiency. Three samples were from CVID patients. (**D**) PCA representing the analysis and comparison of more than 300,000 annotated genes from each group (control and IgA-deficiency samples) recovered by metagenomics and viral metagenomics. Genes were annotated by COG at the IMG-JGI bioinformatic platform. Similar representation was obtained for pfam and other gene annotation methods. (**E**) Massive Metagenomic analysis of pairwise comparison of raw reads obtained from control and IgA-deficiency samples. Metafast program was used to compute the analysis. Heat map illustrate relatedness between the pairwise sample comparison. Color distance from 0 (white color) to value 1 (dark blue) indicates the distance. A value of “0” or white color indicates that two samples are identical. (**F**) Metaviromic analysis of assembled viral contigs from viral metagenomes from groups. Viral metagenomes were quality trimmed, assembled, annotated at the IMG-JGI bioinformatic platform, computed the best-hit scoring for each annotated gene using three different thresholds (30, 60 and 90% of amino acid identity). Bar chart represents the fraction of annotated genes with unknown function (named “unassigned”) using the three different identity thresholds. Data indicate that most of the genes were unknown. Results from the taxonomic assignment of viral genes by best-hit scoring are shown in the pie chart.

In our study, we collected saliva samples from 26 volunteers (16 healthy controls, 7 IgA-deficient patients and 3 with common variable immunodeficiency (CVID) lacking IgA and with altered levels of IgM; in some cases, undetectable) that were processed for 16S rRNA gene Illumina sequencing (Table S1), viral and microbial metagenomics and fluorescence microscopy (Fig. S1). Inclusion criteria were in all cases undetectable seric IgA levels (< 0.07 mg/ml). Patients were recruited from the Primary Immunodeficiencies Unit at Hospital 12 Octubre (Madrid, Spain). Exclusion criteria were antibiotic treatment 6 months before collection and active periodontal/gingival treatments. Clinical data and written consent were obtained from all patients. 16S rRNA gene Illumina sequencing data using amplicon sequence variants (ASV) (proposed to be as reference metric to replace operational taxonomic units in best practices for human microbiome analysis^15^ for unveiling differences in terms of microbial composition^15^) demonstrated that all common commensalistic oral^16^ bacterial genera (e.g. *Streptococcus, Prevotella and Pasteurella*) were predominant in all samples including IgA-deficiency and CVID samples (≥ 80% of total bacterial community, Fig. 1A). Thus, a major microbial shift of bacterial genera was not observed in IgA-deficiency group since all expected typical oral bacteria were detected. Similar data on relative abundance and identity of taxa were obtained from 16S rRNA gene and metagenomics (e.g. taxonomy assignment at the phylum rank, Fig. S2). Slight differences between groups were observed when analyzing the alpha-diversity richness (i.e. total number of ASV) with a moderate/minor decrease of total richness in the IgA-deficiency microbiome (n = 224 ± 44) with lower number of total ASV compared to healthy oral microbiome (n = 263 ± 34.5; Fig. S3, Table S2). This loss of alpha-diversity (≈15%) supported by Faith´s diversity index (p-value 0.02, Fig. 1B) and beta-diversity based on unweighted Unifrac distance metrics (PCoA in Fig. 1C, pairwise Permanova p-value 0.011) is not produced by significant changes in those common, abundant well-known commensal oral bacteria^16^ that were present in all analyzed samples (controls and IgA-deficiency), but it was explained only by a loss of some rare taxa (< 1% relative abundance of each). Rare members of microbiomes sometimes could play key homeostatic roles^17^. However, these differences were not observed by beta-diversity based on weighted Unifrac distance (Fig. 1C, pairwise Permanova p-value 0.093) since relative abundance of dominant bacteria present in both groups (Fig. 1A) likely mask those minor differences in rare abundant bacteria, which overall have a very minor relative contribution to the microbiome composition.

### Microbiome and virome metagenomic analysis

Since differences were observed only for those low abundant taxa between the two groups, we sought then to address whether there was any significant variation on the metabolic capability and functionality of these microbiomes. Diversity and composition changes of human microbiome matters, but what is actually critical is to whether these changes are translated into functional and metabolic shifts that make the microbiome less stable and/or dysfunctional. Metagenomic sequencing data (313,988 and 362,592 annotated genes from controls and Ig-deficient samples, respectively) showed that microbiomes from controls and IgA-deficiency groups have very similar genetic make-up, type and relative proportion of genetic annotated functions (by means of COG, pfam and KO; Fig. 1D, Supplemental Fig. S4) and they therefore clustered together. Indeed, massive pairwise analysis of unassembled raw metagenomic data (50.1 and 44.6 Gb of sequenced reads, Table S3) pointed out to the same outcome (Fig. 1E) since IgA-deficiency samples did not clustered separately from controls. Furthermore, that sample with undetectable levels of both IgA and IgM did not cluster more separately (Fig. 1D, Supplemental Fig. S4). Thus, data suggested that despite IgA deficiency, these microbiomes seem to be as—metabolically—functional as controls.

Furthermore, when we analyzed oral viruses, genetic and functional diversity of oral viruses segregated from that of the oral microbiome (Fig. 1D) indicating that viruses, as previously reported^12,15^, have their own genetic repertoire distinct from that of the oral bacteria and most of the annotated viral genes were unknown (Fig. 1F, bar chart). No apparent differences were observed on viral genetic content between viruses from controls and IgA deficiency group (Fig. 1F). In line with that, metagenomic analyses of taxonomic assignment of predicted viral genes by best-hit scoring assignment using the largest viral database to date (IMG-VR available at JGI-DOE Institute, no of viral genomes > 700,000), showed nearly identical genetic viral information, type of phages and relative abundances of viral genes in both controls and Ig-deficient samples (Fig. 1F). In both groups, common known oral bacterial phages were equally predominant (Fig. 1F).

**Figure 2:**
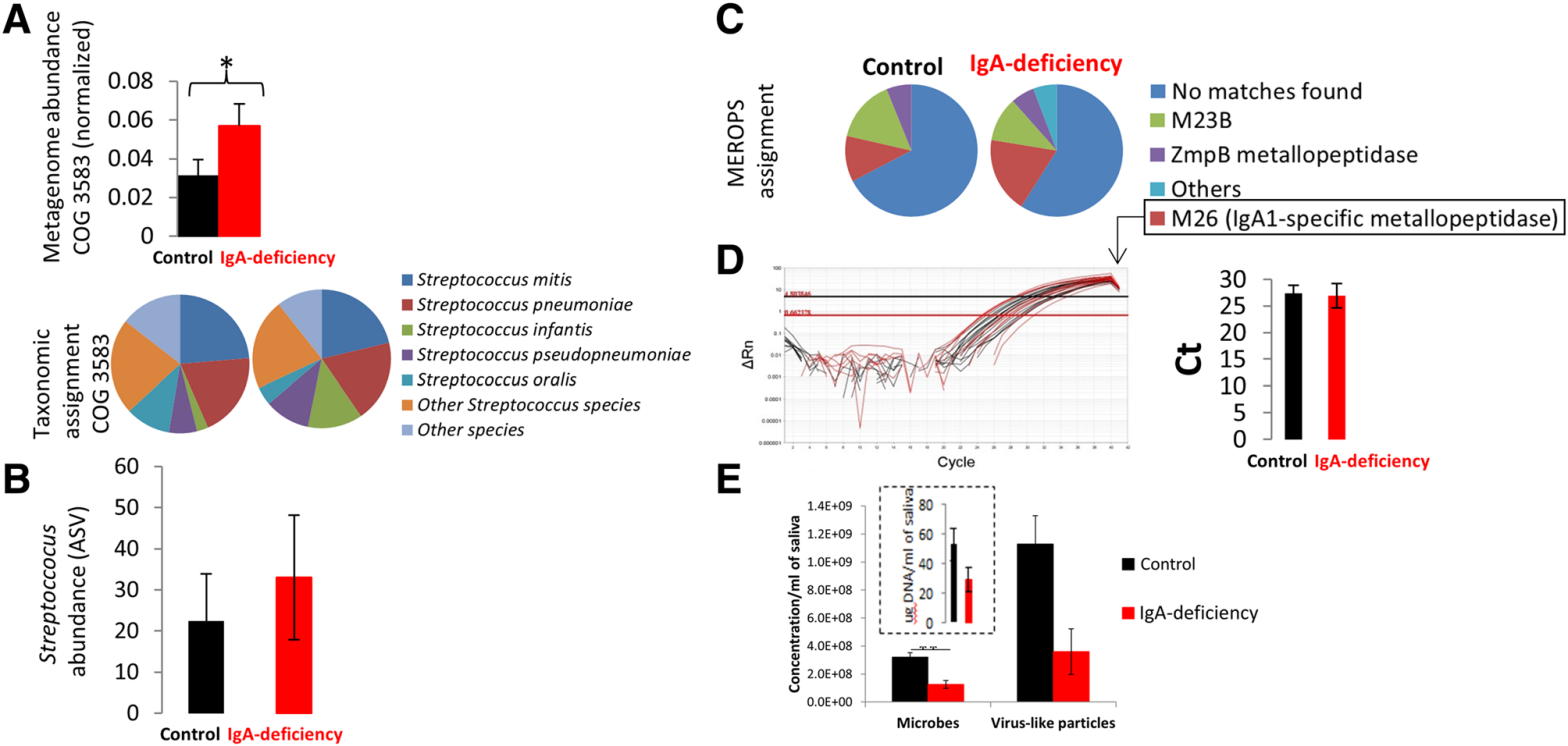
Metagenomic analysis and microbial and viral abundance in controls and IgA deficiency. (**A**) Metagenomic analysis of abundance of genes annotated within COG 3583 involved in the processing and cleavage of IgA (bar chart). An over enrichment is observed from IgA deficiency patients. Taxonomic assignment of bacteria having COG3583 (pie chart) that was clearly dominated by *Streptococcus* spp. (**B**) Relative abundance of amplicon sequence variants (ASV) assigned to genus Streptococcus. Although more *Streptococcus* was found in IgA deficiency samples, differences were not statistically significant. (**C**) Analysis of active domains found in the analyzed proteins belonging to COG3583. MEROPS database was used to identify active domain. IgA metallopeptidases typically have the protease domain M26. (**D**) qPCR results of COG 3583 genes encoding proteases with M26 domain involved in IgA cleavage. No differences in abundance were found between the groups. (**E**) Fluorescence microscopy and microbial and viral abundance obtained by DAPI and SYBR Gold stain, respectively. Small pie chart panel depicts concentration of DNA per ml of saliva obtained from controls and IgA deficiency samples.

Although globally no differences were observed by viral and prokaryote metagenomics between groups, we analyzed whether any particular gene function was over-enriched or depleted in IgA deficiency. Within thousands of the analyzed functions by COG, pfam and KO, we only found a priori differences for the COG3583 (Fig. 2A) with a slight overrepresentation in the Ig-deficiency group (Kruskal–Wallis p-value 0.03 and U-Whitman tests p-value < 0.05). COG3583 includes proteases and proteins involved on processing and cleavage of IgA. It is worth mention that false-discovery rate analysis^18^ by using Benjamin-Hochberg error correction challenged that metagenomic observation on the abundance difference of COG3583 between groups (adjusted p-value 0.63). Taxonomic identification indicated that these COG3583 genes belonged to several oral *Streptococcus* and no differences were observed in relative abundance, taxonomic assignment and type of protease (e.g. metallo-endopeptidase and protease domains M26 potentially involved in IgA processing) between groups (Fig. 2A, pie chart and Fig. 2B, C, Suppl Data S1). In good agreement with the false-discovery rate analysis, we empirically corroborate by qPCR that there was no difference in abundance of COG3583 between both groups (Fig. 2D).

### Microbial and viral abundance in saliva

Remarkably, we did find differences in microbial abundance between groups (Fig. 2E, Kruskal–Wallis p-value 0.0085). Control healthy samples (3.2 × 10^8^ microbes/ml of saliva) approximately displayed two times more cells than Ig-deficient patients (1.3 × 10^8^ microbes/ml of saliva) (Fig. 2E). Accordingly, more total microbial DNA per ml of saliva was also obtained (inner panel in Fig. 2E). Similar abundance trend result was found for oral viruses (Fig. 2E). Previous studies have demonstrated that bacterial phages are enriched in our mucosal surfaces and protects the underlying epithelium from bacterial infection^19^. Enrichment of phage in mucus occurs via binding interactions between mucin glycans and Ig-like protein domains exposed on phage capsids^19^. In the case of IgA-deficiency, it is reasonable to think that lower numbers of anchoring targets (e.g. IgA molecules) are available in our surface barriers for commensal viruses and bacteria.

## Discussion

In this study, a comprehensive 16S rRNA gene and metagenomic analysis of the salivary microbial and viral fractions along with experimental data on viral and microbial abundances was applied. Intriguingly, data indicate that overall microbial and viral diversity and functionality is rather well maintained under IgA-deficiency, even in CVID samples with undetectable levels of IgM and IgA.

The dominant source of the salivary microbiome is most likely bacterial communities on the mucosal surfaces^20^ and it has been demonstrated that IgA not only serve for arresting pathogens at epithelial border but also to bind to commensal oral microbes and anchor them to our mucous barriers^7,8,21^. A priori, according to the reported IgA role^7–9^, more profound changes would be expected in the oral microbiome. It has been proposed that IgM might compensate for a lack of secretory IgA, although recent data challenge that view, since IgM only partially rescue IgA deficiency^11,12^. Here, we have also included three CVID patients (one sample with undetectable IgM levels), which did not segregate more than other IgA-deficiency or control samples (e.g. see Fig. 1C,D). Despite a limiting sampling size in our study, our data showed that when IgA is lacking, and even in CVID, our oral cavity seems to maintain a very similar and functional microbial and viral community albeit lower total microbial abundances are achieved. It is worth noting that the coverage of 16S rRNA gene sequencing data might not be not enough to capture relevant changes on diversity of rare microbes in our samples (i.e. probably abundance lower than 1/1000th of the total community). Overall, our data evidence minimal-moderate changes on oral microbial and viral composition and genetic functionality and then poses the question of: (1) the existence of unknown Ig-independent mechanism/s that aid at shaping commensal bacteria, (2) whether lower abundance of commensal microbes in less stable biofilms might actually matters for homeostasis. Regarding question (1), so far, only the gut microbiome has been investigated in the context of IgA-deficiency and overall dramatic changes have not been observed, with unexpected good levels of typical beneficial symbionts^11^. Our data shed some light into Ig-A deficiency in other niche of our body different than the gut and point to a robust, resilient microbiome and virome. Accumulative recent data suggest that although IgA has an important role for shaping our microbiome^7–9^, it seems not to be critical, since we and others^11^ have observed minimal or moderate changes of our commensal bacteria and viruses. Furthermore, although IgM partially supplies IgA deficiency^11,12^, it does not provided a fully/satisfactory explanation^22^. For instance, IgM does not seem to display the intrinsic high natural polyreactivity of IgA to coat the intestinal microbiota^7^ since the presence of homeostatic secretory IgM (sIgM) responses in the gut was not observable in mouse but sIgM production increases only upon induction of colonic damage^23^. Thus, another explanation to IgA-deficiency not sIgM-dependent could be simply our own microbes, whose resilience and functional redundancy and capacity might compensate these Ig-deficiencies, that even under these stressed conditions, they manage to maintain a rather functional homeostasis “far away” from a severe dysbiosis. In regards to question (2) on lower bacterial and viral commensals, it is important to remark that a denser microbial biofilm of commensal microbes is probably beneficial for our homeostasis preventing the colonization of “foreign unpleased” pathogens and the access of external immunogenic non-biological agents since there is less “free space/gaps” in our surface mucous barriers. Microbes have evolved different strategies to attach to our mucous. In the absence of IgA, there might be an adaptive microbial response, switching the type of expressed adhesins, from a robust IgA-specific adhesion (i.e. “IgA-adhesion lifestyle”) as described for Bacteroides fragilis in the gut^8^, to adhesins targeting other glycans or glycosylated proteins (i.e. “non IgA-adhesion lifestyle”) abundant in our mucous^24^.

In conclusion, this study shows minimal-moderate variations of the microbiome and virome composition in human IgA deficiency in the oral cavity since all common abundant, oral commensalistic microbes were detected with the expected relative abundances. A slight depletion in the alpha richness of rare bacterial taxa was observed in IgA deficiency. Overall, metagenomic and virome data indicated that significant changes in the functional and metabolic capabilities of human oral microbiome and virome were not observed suggesting to a resilient human microbiome and virome under IgA deficiency scenario. Finally, lower number of microbes, statistically supported for bacterial counts, was found in the oral cavity when IgA is absent. It has been recently proposed that glycans in secretory IgA serve as carbon source for our commensal microbes^3^. If so, our data suggest that when IgA is lacking, lower microbial abundances can be sustained, which support the idea that secretory IgA response actually promotes retention of commensal microbes in our mucous than clearance.

## Methods

### Human sample collection and processing for viral and microbial metagenomics

Saliva samples (≈ 5 mL) were obtained from 26 volunteers: 16 healthy controls, 7 IgA-deficient patients and 3 with common variable immunodeficiency (CVID) of IgA, IgG and/or IgM patients). Volunteers signed an informed consent indicating their willingness to participate in this study. This study design and protocols were approved by the Ethic Committee of the University of Alicante within the research project ref. CGL2013-40564-R. None of the subjects or patients included experienced periodontal disease. The patients were randomly selected (53% male and 47% female; age range 11–65) within a cohort of patients with confirmed diagnosis of immunodeficiency at the Primary Immunodeficiency Unit of the Hospital 12 de Octubre (Madrid, Spain). Healthy control samples were collected on 18 January 2016, while samples from IgA deficient patients and CVID were collected on 31 January 2017. In all cases, saliva was collected before breakfast and oral hygiene in the morning and immediately brought to the laboratory on ice. Drinking was not permitted to assess microbial and viral abundance under same conditions. Samples were vortexed for 2 min at maximum speed and then processed for microbial and viral metagenomics and fluorescence microscopy. For microbial metagenomics, 450 ul of samples was stored at − 80 °C until use. DNA extraction was performed with MasterPure™ Complete DNA and RNA Purification Kit (Epibio) following manufacture’s protocol.

For viral metagenomics, DNA extractions were performed within the same day of sample collection as follows. Saliva was centrifuged at 5000×*g* for 10 min at 4 °C and supernatant was sequentially filtered through 0.45 µm and 0.2 µm PES filters (Millipore). Free DNA present in saliva was removed using 50 U of Turbo DNase I (Ambion, Invitrogen) at 37 °C for 1 h. Finally, viral nucleic acids were extracted from 500 ul of DNase treated saliva with QIAmp^®^ UltraSens^®^ Virus Kit (Cat. No 53704, QIAGEN) according to manufacturer’s protocol.

Quality and quantity of extracted DNA from viral and microbial samples were checked with fluorimetry in a Qubit instrument (Invitrogen) and on an electrophoresis gel.

### Sequencing, assembly, annotation and metagenome analyses

Microbial and viral metagenomes were sequenced by Illumina technology using the Nextera XT DNA library (ref. FC-131-1024, Illumina) in a MiSeq sequencer (2 × 250, pair-end) according to manufacturer’s protocol. Raw reads were quality filtered using prinseq-lite program^25^ with the following parameters: min_length: 50, trim_qual_right: 20, trim_qual_type: mean, and trim_qual_window: 20. Additionally, Genome assembly was performed with SPAdes version 3.6.1^26^ using “metaSPADES option” and applying the following parameters: -k 33,55,77,99,127.

General automated annotation was done at the IMG-JGI bioinformatic platform^27^. Manual annotation was also done in house comparing predicted proteins by Prodigal program^28^ with NR database (NCBI) with BLAST version 2.5.0+^29^ and with pfam database using HMMER package^30^. PCA plots of COG, pfam and KO clustering between samples were done with the publicly available bioinformatic tools at IMG-JGI^27^.

Metafast program was used with default parameters to compute pairwise distance for raw read from unassembled metagenomes^31^. Best-score hit analysis with BLAST^29^ of predicted viral genes against > 700,000 viral genomes available at IMGvr database^32^ was performed in house. Genes belonging to COG3583 from metagenomes were downloaded from IMG database and compared against MEROPS database available at EBI-EMBL Institute (https://www.ebi.ac.uk/merops/).

### qPCR experiments of COG3583

Specific primers for the detected genes belonging to COG3583 (n = 254) in microbial metagenomes were designed with Primer 3 program implemented in Geneious bioinformatic package^33^. Two primer sets targeting different gene variants of COG3583 observed after manual alignment were used: primer set 1 197F (5′CAGTCATGGCTGATGGTGCA3′) and 544R (5′CAGTATTCACTGCACGGCT3′), primer set 2 60F (5′AACAGCTGTAACTATGACAGGT3′) and 143R (5′GTTCTAACAGTGTGTGCTGC3′). Real time PCR conditions were as follows: 25 ul final volume reaction with 12.5 ul of 2X Master Mix Power SYBR Green I (Applied Biosystem), 9.5 ul of mQ sterile water, 1 ul of primer forward (10 uM), 1 ul primer reverse (10 uM), 1 ul of DNA template (concentration of 5 ng/ul). Same DNA amount (5 ng in total) was added from all samples to qPCR experiments allowing cross-comparison of Ct. Thermal conditions were as described by manufacture’s protocol.

### 16S rRNA gene sequencing and analysis

PCR of region V4 of 16S rRNA gene and further sequencing was carried out according to Earth Microbiome’s standard protocol with primer set 515F/806R^34^. Sequencing was performed in a Miseq sequencer according to manufacturer’s protocol (pair-end 300 × 2) at the FISABIO Genomics Center (Valencia, Spain). The sequenced data was quality filtered using prinseq-lite with the following parameters min_length: 50, trim_qual_right: 30, trim_qual_type: mean, trim_qual_window: 20 and then joined with FLASH^35^, using default. The primers were removed with cutadapt program, and the cleaned merged reads were analyzed with QIIME2.2020^36^. Low quality reads were eliminated with quality-filter q-score. Deblur^37,38^ was used to trim the sequences at position in order to remove low quality regions.

Diversity was studied using the QIIME2 plugin q2-diversity. Specifically, alpha-diversity was evaluated with Pielou’s Evenness, Shannon’s Diversity index and Faith’s Phylogenetic Diversity index and compared with the no-parametric Kruskal–Wallis test. Beta-diversity was studied using PERMANOVA with the Bray-Courtis distance, Jaccard distance and weighted Unifrac and unweighted Unifrac distances. PCoAs (–p-metric seuclidean) were performed for representing beta-diversity and for all the taxonomic levels, that were previously collapsed. Taxonomy was assigned with the already pre-formatted SILVA 138 database (reproducible sequence taxonomy reference database management for the masses. The comparison between taxa’s relative abundance to find differentially abundant features was performed with ANCOM^39^.

### Fluorescence microscopy and microbial abundance

For microbial counts (see Fig. S1), saliva sample (50 ul) was fixed with glutaraldehyde (0.5% final concentration w/w) at 4 °C for 30 min and store at − 80 °C until use. DAPI stain was performed as described. Briefly, sample was diluted with sterile PBS buffer 1× up to 1 ml and the filtered through 0.2 um GTTP membrane filters (Millipore). For viral counts (see Fig. S1), fixation was as above and sample was stored at -80ºC until use. SYBR Gold stain was performed as described^40,41^.

All methods were carried out in accordance with Spanish and eurpean relevant guidelines and regulations. All experimental protocols were approved by the Ethical Committee of the University of Alicante.

### Public access to metagenomic data and 16S rRNA gene sequences

All detailed information of accession numbers to find our sequencing data generated in this study is provided.

### Ethics and approval and consent to participate

All volunteers signed an informed consent indicating their willingness to participate in this study.

## Supplementary Information

Supplementary Information.

## Data Availability

All data is publicly available. Sequencing data, availability and accession number of assembled metagenomes are all available in Supplementary Material.
