# Supplementary material for: Minimal-moderate variation of human oral virome and microbiome in IgA deficiency
Source: Sci Rep. 2021 Jul 21;11:14913. doi: 10.1038/s41598-021-94507-8 (PMC8295364; doi:10.1038/s41598-021-94507-8)

**Supplementary Material for**

**Minimal-moderate variation of human virome and microbiome in IgA deficiency**

Maria José de la Cruz Peña^1^, Luis Ignacio Gonzalez-Granado^2,3^, Inmaculada Garcia-Heredia^1^, Lucia Maestre Carballa^1^ and Manuel Martinez-Garcia^1^*

^1^Department of Physiology, Genetics, and Microbiology, University of Alicante, Alicante, Spain

## ^2^Primary Immunodeficiencies Unit. Pediatrics. Hospital 12 Octubre. Instituto de Investigación Hospital 12 octubre (imas12). Madrid, Spain

^3^ School of Medicine. Complutense University. Madrid. Spain.

*Correspondence to: [m.martinez@ua.es](mailto:m.martinez@ua.es).

This Suplementary Material includes:

-Section of supplementary material for accessing metagenomic data

-Figures S1-S4

-Tables S1-S3

**Public access to metagenomic data**

Viral and microbial assembled metagenomes generated in this study are publicly available at IMG-JGI (Huntemann *et al.*, 2015) database under the following identification numbers indicated in the table as **IMG genome ID** that can be used for searching in that database. Red colour indicate that these correspond to CVID samples

| **IMG Genome ID** | Genome Name / Sample Name **(MICROBIAL METAGENOMES)** | Genome Size assembled | | | Gene Count assembled | | |
| --- | --- | --- | --- | --- | --- | --- | --- |
| 3300022491 | Human saliva microbial communities from oral cavities of healthy adults from Alicante, Spain - individual 1 | 29111330 | | | 35157 | | |
| 3300022479 | Human saliva microbial communities from oral cavities of healthy adults from Alicante, Spain - individual 10 | 14084879 | | | 17378 | | |
| 3300022480 | Human saliva microbial communities from oral cavities of healthy adults from Alicante, Spain - individual 11 | 5085306 | | | 7156 | | |
| 3300022482 | Human saliva microbial communities from oral cavities of healthy adults from Alicante, Spain - individual 12 | 14438064 | | | 19279 | | |
| 3300022477 | Human saliva microbial communities from oral cavities of healthy adults from Alicante, Spain - individual 14 | 330036 | | | 474 | | |
| 3300022488 | Human saliva microbial communities from oral cavities of healthy adults from Alicante, Spain - individual 15 | 20290665 | | | 25879 | | |
| 3300022489 | Human saliva microbial communities from oral cavities of healthy adults from Alicante, Spain - individual 16 | 30886265 | | | 38153 | | |
| 3300022493 | Human saliva microbial communities from oral cavities of healthy adults from Alicante, Spain - individual 17 | 52354973 | | | 61599 | | |
| 3300022473 | Human saliva microbial communities from oral cavities of healthy adults from Alicante, Spain - individual 2 | 14268001 | | | 17389 | | |
| 3300022484 | Human saliva microbial communities from oral cavities of healthy adults from Alicante, Spain - individual 3 | 19175940 | | | 23458 | | |
| 3300022485 | Human saliva microbial communities from oral cavities of healthy adults from Alicante, Spain - individual 4 | 17441767 | | | 22924 | | |
| 3300022474 | Human saliva microbial communities from oral cavities of healthy adults from Alicante, Spain - individual 6 | 3712523 | | | 5230 | | |
| 3300022486 | Human saliva microbial communities from oral cavities of healthy adults from Alicante, Spain - individual 7 | 23689678 | | | 28123 | | |
| 3300022472 | Human saliva microbial communities from oral cavities of healthy adults from Alicante, Spain - individual 8 | 9671306 | | | 11789 | | |
| 3300022476 | Human saliva microbial communities from oral cavities of IgA immunodeficiency people from Alicante, Spain - individual 1 | 4304770 | | | 5960 | | |
| 3300022478 | Human saliva microbial communities from oral cavities of IgA immunodeficiency people from Alicante, Spain - individual 10 | 6134852 | | | 8428 | | |
| 3300022475 | Human saliva microbial communities from oral cavities of IgA immunodeficiency people from Alicante, Spain - individual 2 | 5166800 | | | 6716 | | |
| 3300022490 | Human saliva microbial communities from oral cavities of IgA immunodeficiency people from Alicante, Spain - individual 3 | 33915936 | | | 38921 | | |
| 3300022483 | Human saliva microbial communities from oral cavities of IgA immunodeficiency people from Alicante, Spain - individual 4 | 19339156 | | | 24447 | | |
| 3300022487 | Human saliva microbial communities from oral cavities of IgA immunodeficiency people from Alicante, Spain - individual 5 | 25053798 | | | 29360 | | |
| 3300022590 | Human saliva microbial communities from oral cavities of IgA immunodeficiency people from Alicante, Spain - individual 6 | 71923080 | | | 85731 | | |
| 3300022495 | Human saliva microbial communities from oral cavities of IgA immunodeficiency people from Alicante, Spain - individual 7 | 58991602 | | | 74509 | | |
| 3300022496 | Human saliva microbial communities from oral cavities of IgA immunodeficiency people from Alicante, Spain - individual 8 | 15318436 | | | 20256 | | |
| 3300022494 | Human saliva microbial communities from oral cavities of IgA immunodeficiency people from Alicante, Spain - individual 9 | 57308440 | | | 68268 | | |
| **IMG Genome ID** | Genome Name / Sample Name **(VIRAL METAGENOMES)** | Genome Size assembled | | Gene Count assembled | | | |
| 3300028515 | Human saliva viral communities from oral cavities of healthy adults from Alicante,Spain - individual 1 | 1702328 | | 2505 | | | |
| 3300028504 | Human saliva viral communities from oral cavities of healthy adults from Alicante,Spain - individual 12 | 267982 | | 401 | | | |
| 3300028513 | Human saliva viral communities from oral cavities of healthy adults from Alicante,Spain - individual 13 | 1323485 | | 1985 | | | |
| 3300028516 | Human saliva viral communities from oral cavities of healthy adults from Alicante,Spain - individual 14 | 1819158 | | 2617 | | | |
| 3300028509 | Human saliva viral communities from oral cavities of healthy adults from Alicante,Spain - individual 15 | 1232109 | | 1874 | | | |
| 3300028541 | Human saliva viral communities from oral cavities of healthy adults from Alicante,Spain - individual 17 | 1699083 | | 2464 | | | |
| 3300028537 | Human saliva viral communities from oral cavities of healthy adults from Alicante,Spain - individual 18 | 1098202 | | 1714 | | | |
| 3300028503 | Human saliva viral communities from oral cavities of healthy adults from Alicante,Spain - individual 3 | 698838 | | 1014 | | | |
| 3300028511 | Human saliva viral communities from oral cavities of healthy adults from Alicante,Spain - individual 4 | 1359060 | | 1870 | | | |
| 3300028519 | Human saliva viral communities from oral cavities of healthy adults from Alicante,Spain - individual 5 | 2863798 | | 4137 | | | |
| 3300028514 | Human saliva viral communities from oral cavities of healthy adults from Alicante,Spain - individual 6 | 1492463 | | 2160 | | | |
| 3300028542 | Human saliva viral communities from oral cavities of healthy adults from Alicante,Spain - individual 7 | 4173712 | | 5869 | | | |
| 3300028538 | Human saliva viral communities from oral cavities of healthy adults from Alicante,Spain - individual 8 | 2141600 | | 2989 | | | |
| 3300028520 | Human saliva viral communities from oral cavities of healthy adults from Alicante,Spain - individual 9 | 2287909 | | 3239 | | | |
| 3300028507 | Human saliva viral communities from oral cavities of IgA immunodeficiency people from Alicante,Spain - individual 1 | 889371 | | 1414 | | | |
| 3300028505 | Human saliva viral communities from oral cavities of IgA immunodeficiency people from Alicante,Spain - individual 10 | 276922 | | 461 | | | |
| 3300028508 | Human saliva viral communities from oral cavities of IgA immunodeficiency people from Alicante,Spain - individual 2 | 1024537 | | 1419 | | | |
| 3300028539 | Human saliva viral communities from oral cavities of IgA immunodeficiency people from Alicante,Spain - individual 3 | 953530 | | 1412 | | | |
| 3300028517 | Human saliva viral communities from oral cavities of IgA immunodeficiency people from Alicante,Spain - individual 4 | 1684016 | | 2537 | | | |
| 3300028506 | Human saliva viral communities from oral cavities of IgA immunodeficiency people from Alicante,Spain - individual 5 | 1152454 | | 1627 | | | |
| 3300028512 | Human saliva viral communities from oral cavities of IgA immunodeficiency people from Alicante,Spain - individual 6 | 1492463 | | 2160 | | | |
| 3300028510 | Human saliva viral communities from oral cavities of IgA immunodeficiency people from Alicante,Spain - individual 7 | 1236571 | | 1855 | | | |
| 3300028518 | Human saliva viral communities from oral cavities of IgA immunodeficiency people from Alicante,Spain - individual 8 | 2141600 | | 2989 | | | |
| 3300028544 | Human saliva viral communities from oral cavities of IgA immunodeficiency people from Alicante,Spain - individual 9 | 1207203 | | 1877 | | | |
|  |  | |  | | |  |  |
| **Public access to 16S rRNA gene sequences. Data is available at NCBI with the following Bioproject ID number PRJNA682933** | | | | | |  |  |

**Supplementary figure**


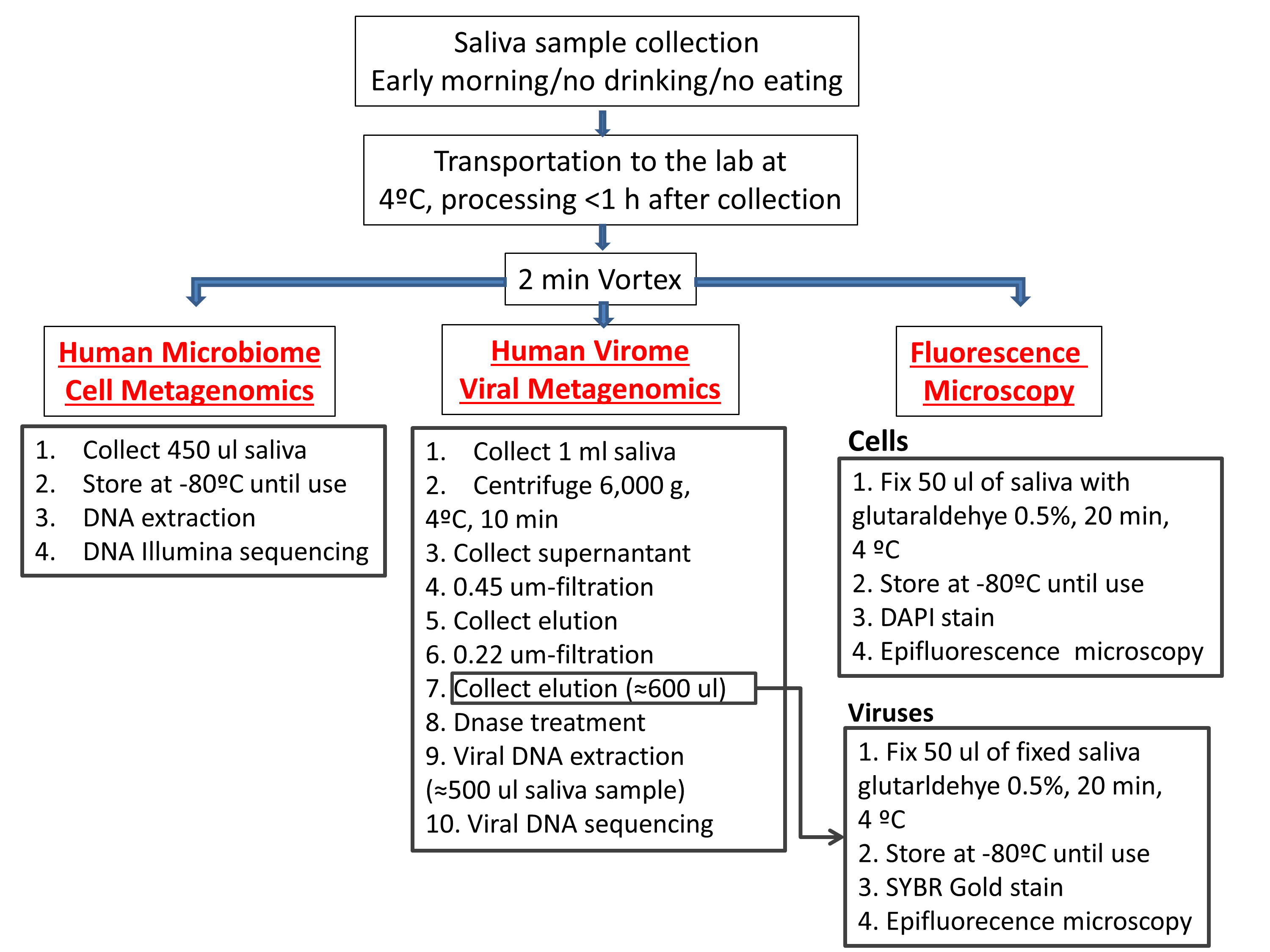


**Fig. S1**. Schematic diagram of saliva sampling and processing


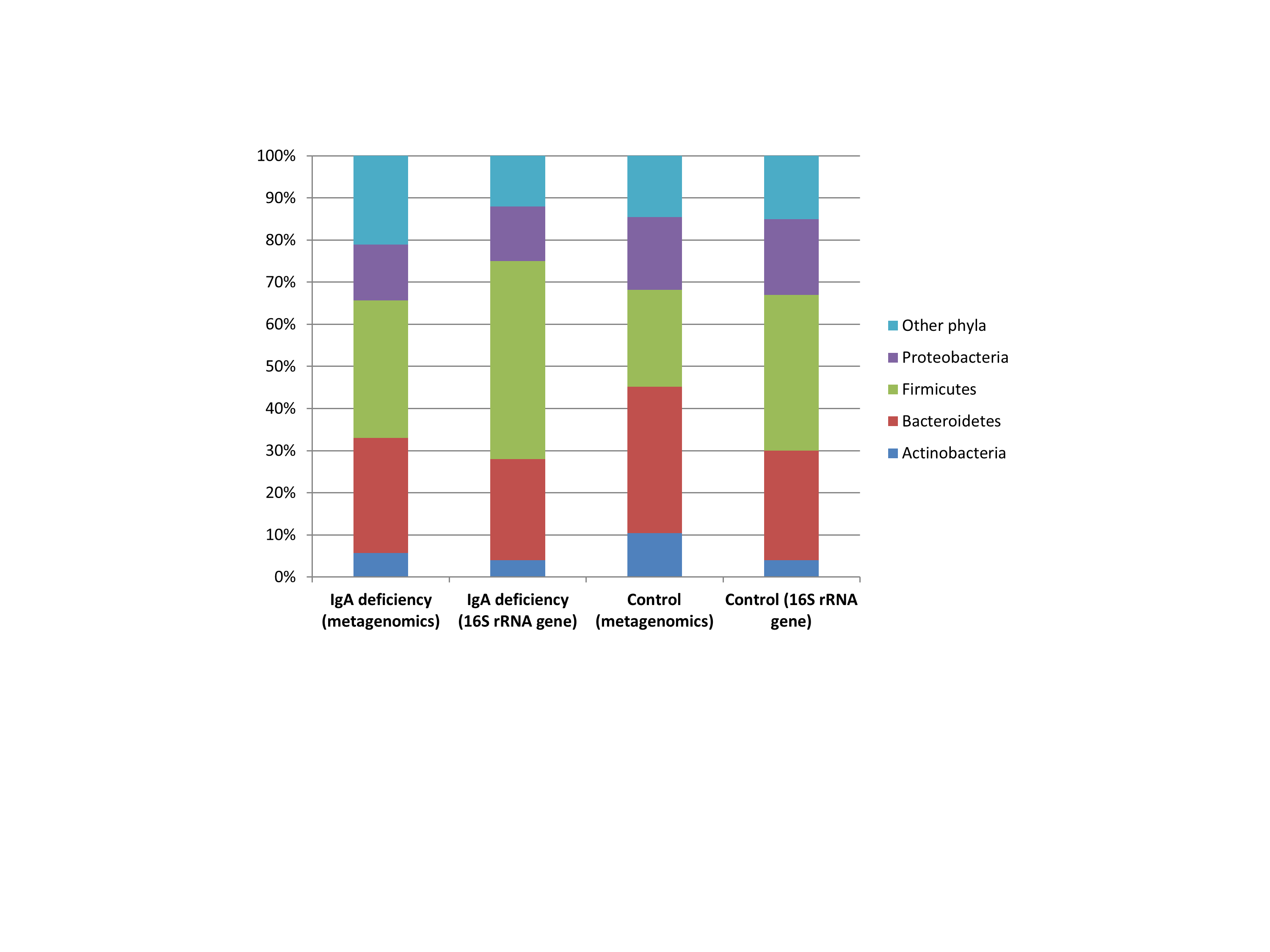


**Fig. S2.** Taxonomic assignment of oral microbiome obtained by metagenomics and 16S rRNA gene analysis. See methods for details. For metagenomics, taxonomic assignment and annotation of genes were carried out at the JGI-IMG annotation server as indicated in Methods.


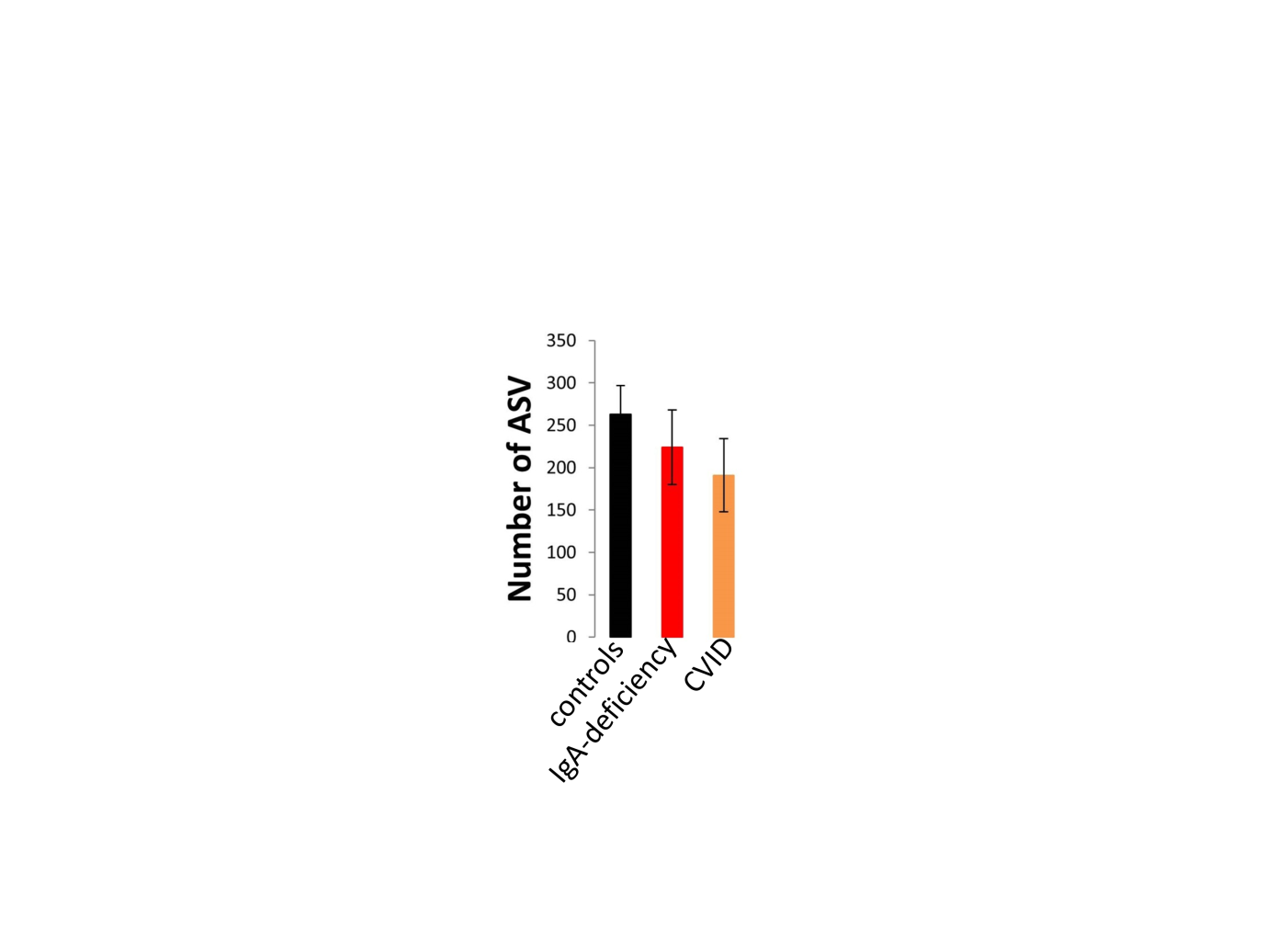


**Fig. S3.** Number of ASV found by Qiime 2 (see method for details) in the analyzed samples. Estimation of difference in alpha richness was then calculated (Fig. 1B).


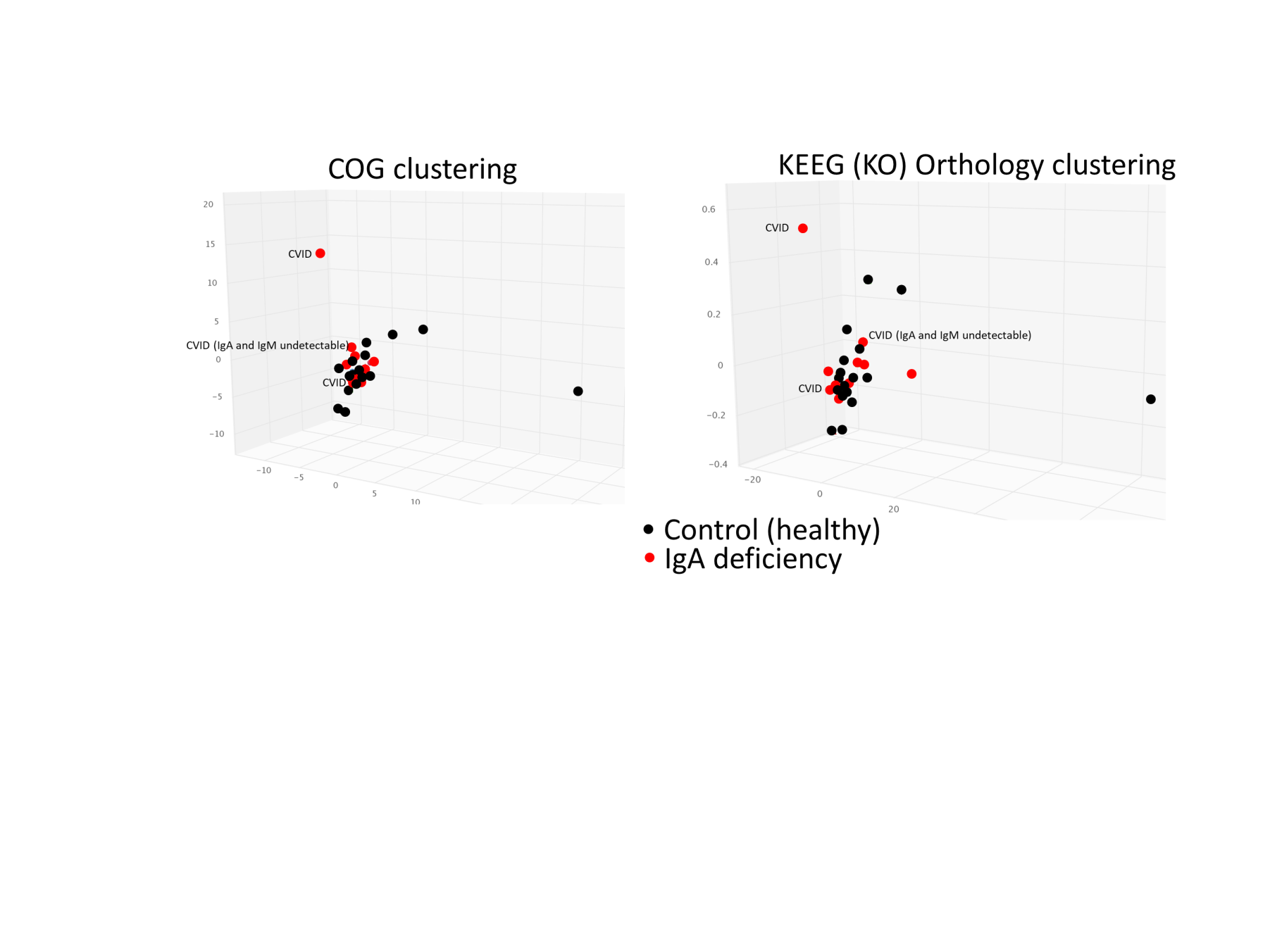


**Fig. S4.** Metagenomic analysis of clustering of samples based on genetic content. ORFs were predicted with Prodigal program at the JGI-IMG platform. Annotation was performed with COG, pfam and KO. All three PCA analyses showed very similar clustering and relatednees between samples. For convenience, pfam clustering is omitted. Note that although two of the analyzed CVID samples were more scattered in the plot, these differences were also observed for some healthy control samples which suggest that natural intrinsic interpersonal variability of our microbimoe might lead these differences.

**Supplementary tables**

| **Table S1: 16S rRNA gene sequences obtained from samples** | | | | | |
| --- | --- | --- | --- | --- | --- |
| **Sample** | **N of reads obtained from Miseq** | **N of reads afer QC** | **N of  joined sequences** | **N q-score filtered sequences** | **N sequences after Deblur Denoising** |
| **Control 1** | 633464 | 623244 | 294723 | 142775 | 3018 |
| **Control 2** | 396814 | 389914 | 184856 | 86364 | 2077 |
| **Control 3** | 348850 | 342288 | 161210 | 68299 | 1793 |
| **Control 4** | 477894 | 469754 | 222635 | 102616 | 2447 |
| **Control 6** | 345280 | 339410 | 160122 | 74284 | 2313 |
| **Control 7** | 452960 | 444614 | 210558 | 94091 | 2295 |
| **Control 8** | 433138 | 425914 | 201444 | 93809 | 2715 |
| **Control 10** | 430134 | 422678 | 200224 | 94355 | 2079 |
| **Control 11** | 510352 | 500930 | 236312 | 103215 | 2688 |
| **Control 12** | 445358 | 437640 | 207009 | 96575 | 2572 |
| **Control 13** | 510566 | 502252 | 237211 | 112758 | 252 |
| **Control 14** | 579006 | 569102 | 269708 | 129915 | 3329 |
| **Control 15** | 464716 | 457618 | 215998 | 92419 | 2281 |
| **Control 16** | 383942 | 378324 | 179562 | 76090 | 1803 |
| **Control 17** | 483166 | 475802 | 225737 | 96942 | 2534 |
| **Control 18** | 477880 | 470934 | 223912 | 96902 | 2738 |
| **IgA1P (CVID)** | 441208 | 434586 | 206552 | 89069 | 2382 |
| **IgA2P (CVID)** | 492388 | 484068 | 229767 | 93570 | 2356 |
| **IgA3P (CVID)** | 466550 | 459434 | 218362 | 95370 | 2817 |
| **IgA4P** | 437314 | 430406 | 204260 | 84459 | 2247 |
| **IgA 5P** | 526896 | 517578 | 244959 | 101682 | 2324 |
| **IgA 6P** | 490422 | 482408 | 228592 | 97855 | 2286 |
| **IgA 7P** | 496122 | 488670 | 231418 | 101961 | 2427 |
| **IgA 8P** | 479434 | 472294 | 224260 | 97478 | 2410 |
| **IgA 9P** | 739212 | 732312 | 350874 | 84839 | 2124 |
| **IgA 10P** | 495638 | 488922 | 233426 | 104812 | 2690 |

**Table S2.** Unique ASV found only in each one of the group. Note that relative abundance for those ASV is extremely low representing thus rare ASV. Taxonomic assignment was done with SILVA database implemented in Qiime2.

| **ASV FOUND ONLY IN IgA patients** | **Mean relative abundance** |
| --- | --- |
| d__Bacteria; p__Bacteroidota; c__Bacteroidia; o__Bacteroidales; f__Porphyromonadaceae; g__Porphyromonas | 0,04797593 |
| d__Bacteria; p__Fusobacteriota; c__Fusobacteriia; o__Fusobacteriales; f__Leptotrichiaceae; g__Leptotrichia; s__Leptotrichia_sp._oral_clone_EI013 | 0,05686789 |
| d__Bacteria; p__Bacteroidota; c__Bacteroidia; o__Flavobacteriales; f__Weeksellaceae; g__Bergeyella; s__uncultured_bacterium | 0,05947955 |
| d__Bacteria; p__Fusobacteriota; c__Fusobacteriia; o__Fusobacteriales; f__Leptotrichiaceae; g__Leptotrichia; s__Leptotrichia_sp._oral_clone_EI013 | 0,0743657 |
| d__Bacteria; p__Fusobacteriota; c__Fusobacteriia; o__Fusobacteriales; f__Leptotrichiaceae; g__Streptobacillus; s__Streptobacillus_hongkongensis | 0,05576208 |
| d__Bacteria; p__Bacteroidota; c__Bacteroidia; o__Bacteroidales; f__Prevotellaceae; g__Alloprevotella | 0,06899409 |
| d__Bacteria; p__Proteobacteria; c__Gammaproteobacteria; o__Burkholderiales; f__Neisseriaceae; g__Neisseria | 0,07209778 |
| d__Bacteria; p__Bacteroidota; c__Bacteroidia; o__Bacteroidales; f__Prevotellaceae; g__Prevotella; s__Prevotella_sp._oral_taxon_299_str._F0039 | 0,04250356 |
| d__Bacteria; p__Firmicutes; c__Clostridia; o__Peptostreptococcales-Tissierellales; f__Peptostreptococcales-Tissierellales; g__Parvimonas | 0,05037783 |
| d__Bacteria; p__Fusobacteriota; c__Fusobacteriia; o__Fusobacteriales; f__Fusobacteriaceae; g__Fusobacterium | 0,04622103 |
| d__Bacteria; p__Bacteroidota; c__Bacteroidia; o__Bacteroidales; f__Porphyromonadaceae; g__Porphyromonas | 0,10828625 |
| d__Bacteria; p__Patescibacteria; c__Gracilibacteria; o__Absconditabacteriales_(SR1); f__Absconditabacteriales_(SR1); g__Absconditabacteriales_(SR1) | 0,05873396 |
| d__Bacteria; p__Fusobacteriota; c__Fusobacteriia; o__Fusobacteriales; f__Leptotrichiaceae; g__Leptotrichia | 0,05204028 |
| d__Bacteria; p__Fusobacteriota; c__Fusobacteriia; o__Fusobacteriales; f__Leptotrichiaceae; g__Streptobacillus; s__uncultured_bacterium | 0,04089219 |
| d__Bacteria; p__Bacteroidota; c__Bacteroidia; o__Bacteroidales; f__Porphyromonadaceae; g__Porphyromonas | 0,05249344 |
| d__Bacteria; p__Bacteroidota; c__Bacteroidia; o__Bacteroidales; f__Prevotellaceae; g__Prevotella | 0,04783288 |
| d__Bacteria; p__Proteobacteria; c__Gammaproteobacteria; o__Pasteurellales; f__Pasteurellaceae; g__Haemophilus | 0,12274643 |
| d__Bacteria; p__Bacteroidota; c__Bacteroidia; o__Bacteroidales; f__Porphyromonadaceae; g__Porphyromonas; s__Porphyromonas_sp._oral_clone_DP023 | 0,05024276 |
| d__Bacteria; p__Bacteroidota; c__Bacteroidia; o__Bacteroidales; f__Prevotellaceae; g__Prevotella | 0,0813365 |
| d__Bacteria; p__Fusobacteriota; c__Fusobacteriia; o__Fusobacteriales; f__Leptotrichiaceae; g__Leptotrichia; s__Leptotrichia_sp._oral_clone_EI013 | 0,19247594 |
| d__Bacteria; p__Bacteroidota; c__Bacteroidia; o__Bacteroidales; f__Prevotellaceae; g__Alloprevotella | 0,09888752 |
| d__Bacteria; p__Firmicutes; c__Bacilli; o__Lactobacillales | 0,03717472 |
| d__Bacteria; p__Bacteroidota; c__Bacteroidia; o__Bacteroidales; f__Prevotellaceae; g__Prevotella; s__Prevotella_pleuritidis | 0,04198153 |
| d__Bacteria; p__Proteobacteria; c__Gammaproteobacteria; o__Pasteurellales; f__Pasteurellaceae; g__Actinobacillus | 0,13796173 |
| d__Bacteria; p__Firmicutes; c__Bacilli; o__Lactobacillales | 0,04730384 |
| d__Bacteria; p__Proteobacteria; c__Gammaproteobacteria; o__Pasteurellales; f__Pasteurellaceae | 0,03717472 |
| d__Bacteria; p__Fusobacteriota; c__Fusobacteriia; o__Fusobacteriales; f__Leptotrichiaceae; g__Streptobacillus; s__uncultured_bacterium | 0,06609556 |
| d__Bacteria; p__Firmicutes; c__Bacilli; o__Lactobacillales | 0,04855155 |
| d__Bacteria; p__Patescibacteria; c__Gracilibacteria; o__Absconditabacteriales_(SR1); f__Absconditabacteriales_(SR1); g__Absconditabacteriales_(SR1) | 0,03717472 |
| d__Bacteria; p__Bacteroidota; c__Bacteroidia; o__Bacteroidales; f__Prevotellaceae; g__Prevotella | 0,0713686 |
| d__Bacteria; p__Patescibacteria; c__Gracilibacteria; o__Absconditabacteriales_(SR1); f__Absconditabacteriales_(SR1); g__Absconditabacteriales_(SR1) | 0,04460967 |
| d__Bacteria; p__Bacteroidota; c__Bacteroidia; o__Bacteroidales; f__Prevotellaceae; g__Prevotella; s__Prevotella_sp._HJM029 | 0,0521358 |
| d__Bacteria; p__Bacteroidota; c__Bacteroidia; o__Flavobacteriales; f__Weeksellaceae; g__Bergeyella; s__uncultured_bacterium | 0,03717472 |
| d__Bacteria; p__Actinobacteriota; c__Actinobacteria; o__Micrococcales; f__Micrococcaceae; g__Rothia; s__uncultured_organism | 0,03904863 |
| d__Bacteria; p__Bacteroidota; c__Bacteroidia; o__Bacteroidales; f__Porphyromonadaceae; g__Porphyromonas | 0,12141992 |
| d__Bacteria; p__Firmicutes; c__Negativicutes; o__Veillonellales-Selenomonadales; f__Veillonellaceae; g__Veillonella | 0,07428563 |
| d__Bacteria; p__Fusobacteriota; c__Fusobacteriia; o__Fusobacteriales; f__Leptotrichiaceae; g__Leptotrichia; s__Leptotrichia_sp._oral_clone_EI013 | 0,04804746 |
| d__Bacteria; p__Proteobacteria; c__Gammaproteobacteria; o__Pasteurellales; f__Pasteurellaceae; g__Haemophilus | 0,05785295 |
| d__Bacteria; p__Proteobacteria; c__Gammaproteobacteria; o__Pasteurellales; f__Pasteurellaceae; g__Haemophilus | 0,04978711 |
| d__Bacteria; p__Bacteroidota; c__Bacteroidia; o__Bacteroidales; f__Prevotellaceae; g__Prevotella; s__Prevotella_sp._GEJ23 | 0,04733219 |
| d__Bacteria; p__Bacteroidota; c__Bacteroidia; o__Bacteroidales; f__Prevotellaceae; g__Prevotella | 0,18272081 |
| d__Bacteria; p__Fusobacteriota; c__Fusobacteriia; o__Fusobacteriales; f__Leptotrichiaceae; g__Streptobacillus; s__uncultured_bacterium | 0,04129503 |
| d__Bacteria; p__Firmicutes; c__Negativicutes; o__Veillonellales-Selenomonadales; f__Veillonellaceae; g__Veillonella | 0,12911846 |
| d__Bacteria; p__Bacteroidota; c__Bacteroidia; o__Bacteroidales; f__Prevotellaceae; g__Alloprevotella | 0,04532344 |
| d__Bacteria; p__Patescibacteria; c__Gracilibacteria; o__Absconditabacteriales_(SR1); f__Absconditabacteriales_(SR1); g__Absconditabacteriales_(SR1) | 0,04089219 |
| d__Bacteria; p__Bacteroidota; c__Bacteroidia; o__Bacteroidales; f__Porphyromonadaceae; g__Porphyromonas | 0,04305868 |
| d__Bacteria; p__Bacteroidota; c__Bacteroidia; o__Bacteroidales; f__Prevotellaceae; g__Prevotella; s__Prevotella_pleuritidis | 0,07990389 |
| d__Bacteria; p__Bacteroidota; c__Bacteroidia; o__Bacteroidales; f__Prevotellaceae; g__Prevotella; s__Prevotella_pleuritidis | 0,04641133 |
| d__Bacteria; p__Actinobacteriota; c__Actinobacteria; o__Micrococcales; f__Micrococcaceae; g__Rothia; s__uncultured_organism | 0,03904863 |
| d__Bacteria; p__Bacteroidota; c__Bacteroidia; o__Bacteroidales; f__Prevotellaceae; g__Prevotella | 0,06024096 |
| d__Bacteria; p__Bacteroidota; c__Bacteroidia; o__Bacteroidales; f__Porphyromonadaceae; g__Porphyromonas | 0,12183483 |
| d__Bacteria; p__Fusobacteriota; c__Fusobacteriia; o__Fusobacteriales; f__Fusobacteriaceae; g__Fusobacterium | 0,05517827 |
| d__Bacteria; p__Fusobacteriota; c__Fusobacteriia; o__Fusobacteriales; f__Leptotrichiaceae; g__Leptotrichia; s__Leptotrichia_sp._oral_clone_EI013 | 0,15310586 |
| d__Bacteria; p__Firmicutes; c__Bacilli; o__Staphylococcales; f__Gemellaceae; g__Gemella | 0,04115793 |
| d__Bacteria; p__Fusobacteriota; c__Fusobacteriia; o__Fusobacteriales; f__Leptotrichiaceae; g__Leptotrichia; s__Leptotrichia_sp._oral_clone_EI013 | 0,31496063 |
| d__Bacteria; p__Fusobacteriota; c__Fusobacteriia; o__Fusobacteriales; f__Fusobacteriaceae; g__Fusobacterium | 0,0466893 |
| d__Bacteria; p__Proteobacteria; c__Gammaproteobacteria; o__Pasteurellales; f__Pasteurellaceae; g__Haemophilus | 0,04270476 |
| d__Bacteria; p__Fusobacteriota; c__Fusobacteriia; o__Fusobacteriales; f__Leptotrichiaceae; g__Streptobacillus; s__uncultured_bacterium | 0,07724167 |
| d__Bacteria; p__Bacteroidota; c__Bacteroidia; o__Bacteroidales; f__Prevotellaceae; g__Alloprevotella | 0,09064689 |
| d__Bacteria; p__Patescibacteria; c__Gracilibacteria; o__Absconditabacteriales_(SR1); f__Absconditabacteriales_(SR1); g__Absconditabacteriales_(SR1) | 0,07806691 |
| d__Bacteria; p__Fusobacteriota; c__Fusobacteriia; o__Fusobacteriales; f__Leptotrichiaceae; g__Streptobacillus; s__uncultured_bacterium | 0,05178439 |
| d__Bacteria; p__Firmicutes; c__Bacilli; o__Lactobacillales; f__Streptococcaceae; g__Streptococcus | 0,04259851 |
| d__Bacteria; p__Fusobacteriota; c__Fusobacteriia; o__Fusobacteriales; f__Fusobacteriaceae; g__Fusobacterium | 0,05897546 |
| d__Bacteria; p__Firmicutes; c__Bacilli; o__Lactobacillales; f__Streptococcaceae; g__Streptococcus | 0,19426314 |
| d__Bacteria; p__Bacteroidota; c__Bacteroidia; o__Bacteroidales; f__Prevotellaceae; g__Alloprevotella; s__Prevotella_sp. | 0,04360078 |

| **ASV FOUND ONLY IN COTROLS** | **Mean relative abundance** |
| --- | --- |
| d__Bacteria; p__Fusobacteriota; c__Fusobacteriia; o__Fusobacteriales; f__Leptotrichiaceae; g__uncultured;Ambiguous_taxa | 0,01877441 |
| d__Bacteria; p__Fusobacteriota; c__Fusobacteriia; o__Fusobacteriales; f__Fusobacteriaceae; g__Fusobacterium | 0,01959908 |
| d__Bacteria; p__Proteobacteria; c__Gammaproteobacteria; o__Pasteurellales; f__Pasteurellaceae | 0,02065185 |
| d__Bacteria; p__Bacteroidota; c__Bacteroidia; o__Bacteroidales; f__Prevotellaceae; g__Prevotella; s__Prevotella_veroralis | 0,02070908 |
| d__Bacteria; p__Actinobacteriota; c__Coriobacteriia; o__Coriobacteriales; f__Atopobiaceae; g__Atopobium; s__uncultured_organism | 0,02070908 |
| d__Bacteria; p__Proteobacteria; c__Gammaproteobacteria; o__Pasteurellales; f__Pasteurellaceae | 0,02252929 |
| d__Bacteria; p__Proteobacteria; c__Gammaproteobacteria; o__Pasteurellales; f__Pasteurellaceae | 0,02282688 |
| d__Bacteria; p__Firmicutes; c__Bacilli; o__Erysipelotrichales; f__Erysipelotrichaceae; g__Solobacterium; s__Solobacterium_moorei | 0,02290685 |
| d__Bacteria; p__Bacteroidota; c__Bacteroidia; o__Bacteroidales; f__Prevotellaceae; g__Alloprevotella; s__Prevotella_sp. | 0,02302026 |
| d__Bacteria; p__Firmicutes; c__Bacilli; o__Lactobacillales; f__Streptococcaceae; g__Streptococcus | 0,02302026 |
| d__Bacteria; p__Bacteroidota; c__Bacteroidia; o__Bacteroidales; f__Prevotellaceae; g__Prevotella; s__Prevotella_sp._oral_taxon_299_str._F0039 | 0,02302026 |
| d__Bacteria; p__Firmicutes; c__Bacilli; o__Lactobacillales | 0,02316272 |
| d__Bacteria; p__Bacteroidota; c__Bacteroidia; o__Bacteroidales; f__Prevotellaceae; g__Alloprevotella; s__Prevotella_sp. | 0,02351355 |
| d__Bacteria; p__Firmicutes; c__Clostridia; o__Peptococcales; f__Peptococcaceae; g__Peptococcus;Ambiguous_taxa | 0,02382984 |
| d__Bacteria; p__Bacteroidota; c__Bacteroidia; o__Bacteroidales; f__Prevotellaceae; g__Prevotella; s__Prevotella_sp._oral_taxon_299_str._F0039 | 0,02389626 |
| d__Bacteria; p__Campilobacterota; c__Campylobacteria; o__Campylobacterales; f__Campylobacteraceae; g__Campylobacter | 0,02393549 |
| d__Bacteria; p__Firmicutes; c__Clostridia; o__Lachnospirales; f__Lachnospiraceae; g__Oribacterium | 0,0239499 |
| d__Bacteria; p__Bacteroidota; c__Bacteroidia; o__Bacteroidales; f__Porphyromonadaceae; g__Porphyromonas; s__unidentified | 0,02424084 |
| d__Bacteria; p__Firmicutes; c__Negativicutes; o__Veillonellales-Selenomonadales; f__Veillonellaceae; g__Veillonella | 0,02448236 |
| d__Bacteria; p__Bacteroidota; c__Bacteroidia; o__Bacteroidales; f__Prevotellaceae; g__Prevotella; s__Prevotella_nigrescens | 0,02455106 |
| d__Bacteria; p__Bacteroidota; c__Bacteroidia; o__Bacteroidales; f__Prevotellaceae; g__Prevotella; s__Prevotella_pallens | 0,02466456 |
| d__Bacteria; p__Patescibacteria; c__Gracilibacteria; o__Absconditabacteriales_(SR1); f__Absconditabacteriales_(SR1); g__Absconditabacteriales_(SR1); s__SR1_bacterium_oral_taxon_875 | 0,02466456 |
| d__Bacteria; p__Firmicutes; c__Bacilli; o__Lactobacillales; f__Streptococcaceae; g__Streptococcus | 0,02485089 |
| d__Bacteria; p__Firmicutes; c__Bacilli; o__Staphylococcales; f__Gemellaceae; g__Gemella | 0,02494957 |
| d__Bacteria; p__Firmicutes; c__Clostridia; o__Lachnospirales; f__Lachnospiraceae; g__Oribacterium | 0,0253033 |
| d__Bacteria; p__Firmicutes; c__Negativicutes; o__Veillonellales-Selenomonadales; f__Veillonellaceae; g__Veillonella | 0,02554148 |
| d__Bacteria; p__Proteobacteria; c__Gammaproteobacteria; o__Burkholderiales; f__Neisseriaceae | 0,02554148 |
| d__Bacteria; p__Firmicutes; c__Bacilli; o__Lactobacillales; f__Streptococcaceae | 0,02556973 |
| d__Bacteria; p__Firmicutes; c__Bacilli; o__Lactobacillales | 0,02559423 |
| d__Bacteria; p__Firmicutes; c__Negativicutes; o__Veillonellales-Selenomonadales; f__Veillonellaceae; g__Veillonella | 0,02566074 |
| d__Bacteria; p__Bacteroidota; c__Bacteroidia; o__Flavobacteriales; f__Weeksellaceae; g__Bergeyella; s__uncultured_Bergeyella_sp. | 0,02569897 |
| d__Bacteria; p__Bacteroidota; c__Bacteroidia; o__Bacteroidales; f__Prevotellaceae; g__Alloprevotella; s__Prevotella_sp. | 0,02588666 |
| d__Bacteria; p__Bacteroidota; c__Bacteroidia; o__Bacteroidales; f__Prevotellaceae; g__Prevotella; s__Prevotella_nigrescens | 0,02596609 |
| d__Bacteria; p__Actinobacteriota; c__Coriobacteriia; o__Coriobacteriales; f__Atopobiaceae; g__Atopobium | 0,02611709 |
| d__Bacteria; p__Firmicutes; c__Negativicutes; o__Veillonellales-Selenomonadales; f__Veillonellaceae; g__Veillonella | 0,02617574 |
| d__Bacteria; p__Fusobacteriota; c__Fusobacteriia; o__Fusobacteriales; f__Leptotrichiaceae; g__uncultured;Ambiguous_taxa | 0,02628417 |
| d__Bacteria; p__Fusobacteriota; c__Fusobacteriia; o__Fusobacteriales; f__Fusobacteriaceae; g__Fusobacterium | 0,02629264 |
| d__Bacteria; p__Bacteroidota; c__Bacteroidia; o__Bacteroidales; f__Prevotellaceae; g__Alloprevotella | 0,02649186 |
| d__Bacteria; p__Firmicutes; c__Negativicutes; o__Veillonellales-Selenomonadales; f__Selenomonadaceae; g__Selenomonas | 0,02649418 |
| d__Bacteria; p__Firmicutes; c__Clostridia; o__Peptostreptococcales-Tissierellales; f__Anaerovoracaceae; g__[Eubacterium]_nodatum_group; s__[Eubacterium]_infirmum | 0,02655637 |
| d__Bacteria; p__Firmicutes; c__Negativicutes; o__Veillonellales-Selenomonadales; f__Veillonellaceae; g__Veillonella | 0,02657151 |
| d__Bacteria; p__Fusobacteriota; c__Fusobacteriia; o__Fusobacteriales; f__Leptotrichiaceae; g__Leptotrichia | 0,02665675 |
| d__Bacteria; p__Campilobacterota; c__Campylobacteria; o__Campylobacterales; f__Campylobacteraceae; g__Campylobacter; s__uncultured_organism | 0,02666382 |
| d__Bacteria; p__Proteobacteria; c__Gammaproteobacteria; o__Burkholderiales; f__Neisseriaceae; g__Neisseria; s__Neisseria_flavescens | 0,02673017 |
| d__Bacteria; p__Fusobacteriota; c__Fusobacteriia; o__Fusobacteriales; f__Fusobacteriaceae; g__Fusobacterium | 0,0268634 |
| d__Bacteria; p__Bacteroidota; c__Bacteroidia; o__Flavobacteriales; f__Weeksellaceae; g__Bergeyella; s__uncultured_Bergeyella_sp. | 0,02698296 |
| d__Bacteria; p__Bacteroidota; c__Bacteroidia; o__Bacteroidales; f__Porphyromonadaceae; g__Porphyromonas; s__Porphyromonas_gingivalis | 0,02702118 |
| d__Bacteria; p__Firmicutes; c__Bacilli; o__Mycoplasmatales; f__Mycoplasmataceae; g__Mycoplasma | 0,02702118 |
| d__Bacteria; p__Fusobacteriota; c__Fusobacteriia; o__Fusobacteriales; f__Fusobacteriaceae; g__Fusobacterium | 0,02705005 |
| d__Bacteria; p__Fusobacteriota; c__Fusobacteriia; o__Fusobacteriales; f__Fusobacteriaceae; g__Fusobacterium | 0,02705918 |
| d__Bacteria; p__Firmicutes; c__Negativicutes; o__Veillonellales-Selenomonadales; f__Veillonellaceae; g__Veillonella | 0,02728175 |
| d__Bacteria; p__Bacteroidota; c__Bacteroidia; o__Bacteroidales; f__Prevotellaceae; g__Prevotella; s__Prevotella_veroralis | 0,02734681 |
| d__Bacteria; p__Bacteroidota; c__Bacteroidia; o__Bacteroidales; f__Prevotellaceae; g__Prevotella; s__Prevotella_sp._GEJ23 | 0,02735181 |
| d__Bacteria; p__Bacteroidota; c__Bacteroidia; o__Bacteroidales; f__Porphyromonadaceae; g__Porphyromonas; s__Porphyromonas_sp._oral_clone_DP023 | 0,02743376 |
| d__Bacteria; p__Bacteroidota; c__Bacteroidia; o__Flavobacteriales; f__Weeksellaceae; g__Bergeyella; s__uncultured_Bergeyella_sp. | 0,02754276 |
| d__Bacteria; p__Bacteroidota; c__Bacteroidia; o__Bacteroidales; f__Prevotellaceae; g__Alloprevotella; s__Prevotella_sp. | 0,02755977 |
| d__Bacteria; p__Actinobacteriota; c__Actinobacteria; o__Micrococcales; f__Micrococcaceae; g__Rothia; s__uncultured_bacterium | 0,02763431 |
| d__Bacteria; p__Bacteroidota; c__Bacteroidia; o__Bacteroidales; f__Prevotellaceae; g__Prevotella; s__Prevotella_sp._oral_taxon_299_str._F0039 | 0,02763758 |
| d__Bacteria; p__Fusobacteriota; c__Fusobacteriia; o__Fusobacteriales; f__Fusobacteriaceae; g__Fusobacterium | 0,02767008 |
| d__Bacteria; p__Firmicutes; c__Negativicutes; o__Veillonellales-Selenomonadales; f__Veillonellaceae; g__Veillonella | 0,02790179 |
| d__Bacteria; p__Bacteroidota; c__Bacteroidia; o__Bacteroidales; f__Porphyromonadaceae; g__Porphyromonas | 0,02805921 |
| d__Bacteria; p__Proteobacteria; c__Gammaproteobacteria; o__Pasteurellales; f__Pasteurellaceae; g__Actinobacillus | 0,02809563 |
| d__Bacteria; p__Bacteroidota; c__Bacteroidia; o__Bacteroidales; f__Prevotellaceae; g__Prevotella; s__Prevotella_pallens | 0,02859687 |
| d__Bacteria; p__Firmicutes; c__Bacilli; o__Staphylococcales; f__Gemellaceae; g__Gemella | 0,02878364 |
| d__Bacteria; p__Bacteroidota; c__Bacteroidia; o__Bacteroidales; f__Prevotellaceae; g__Prevotella; s__Prevotella_pallens | 0,02922248 |
| d__Bacteria; p__Firmicutes; c__Bacilli; o__Lactobacillales; f__Streptococcaceae | 0,02938146 |
| d__Bacteria; p__Fusobacteriota; c__Fusobacteriia; o__Fusobacteriales; f__Leptotrichiaceae; g__uncultured;Ambiguous_taxa | 0,02946218 |
| d__Bacteria; p__Campilobacterota; c__Campylobacteria; o__Campylobacterales; f__Campylobacteraceae; g__Campylobacter | 0,02959747 |
| d__Bacteria; p__Firmicutes; c__Negativicutes; o__Veillonellales-Selenomonadales; f__Veillonellaceae; g__Megasphaera; s__Megasphaera_micronuciformis | 0,02963785 |
| d__Bacteria; p__Proteobacteria; c__Gammaproteobacteria; o__Pasteurellales; f__Pasteurellaceae | 0,02971288 |
| d__Bacteria; p__Firmicutes; c__Negativicutes; o__Veillonellales-Selenomonadales; f__Veillonellaceae; g__Veillonella | 0,02971327 |
| d__Bacteria; p__Firmicutes; c__Clostridia; o__Peptostreptococcales-Tissierellales; f__Anaerovoracaceae; g__[Eubacterium]_nodatum_group; s__[Eubacterium]_infirmum | 0,02975154 |
| d__Bacteria; p__Firmicutes; c__Bacilli; o__Lactobacillales; f__Streptococcaceae; g__Streptococcus | 0,0299263 |
| d__Bacteria; p__Firmicutes; c__Bacilli; o__Lactobacillales; f__Streptococcaceae; g__Streptococcus | 0,02995643 |
| d__Bacteria; p__Bacteroidota; c__Bacteroidia; o__Bacteroidales; f__Prevotellaceae; g__Prevotella; s__Prevotella_veroralis | 0,03005161 |
| d__Bacteria; p__Bacteroidota; c__Bacteroidia; o__Bacteroidales; f__Prevotellaceae; g__Prevotella; s__Prevotella_sp._GEJ23 | 0,03006253 |
| d__Bacteria; p__Firmicutes; c__Clostridia; o__Peptostreptococcales-Tissierellales; f__Peptostreptococcaceae; g__Filifactor;Ambiguous_taxa | 0,03006253 |
| d__Bacteria; p__Firmicutes; c__Negativicutes; o__Veillonellales-Selenomonadales; f__Veillonellaceae; g__Veillonella | 0,03008279 |
| d__Bacteria; p__Bacteroidota; c__Bacteroidia; o__Bacteroidales; f__Prevotellaceae; g__Alloprevotella; s__Prevotella_sp._oral_clone_BU035 | 0,03009148 |
| d__Bacteria; p__Bacteroidota; c__Bacteroidia; o__Bacteroidales; f__Prevotellaceae; g__Alloprevotella; s__Prevotella_sp._oral_clone_BU035 | 0,03009148 |
| d__Bacteria; p__Bacteroidota; c__Bacteroidia; o__Bacteroidales; f__Prevotellaceae; g__Alloprevotella; s__Prevotella_sp._oral_clone_BU035 | 0,03009148 |
| d__Bacteria; p__Firmicutes; c__Bacilli; o__Lactobacillales; f__Streptococcaceae; g__Streptococcus | 0,03014029 |
| d__Bacteria; p__Firmicutes; c__Bacilli; o__Lactobacillales; f__Streptococcaceae; g__Streptococcus | 0,03014029 |
| d__Bacteria; p__Firmicutes; c__Bacilli; o__Lactobacillales | 0,03020531 |
| d__Bacteria; p__Bacteroidota; c__Bacteroidia; o__Flavobacteriales; f__Weeksellaceae; g__Bergeyella; s__uncultured_bacterium | 0,03023354 |
| d__Bacteria; p__Fusobacteriota; c__Fusobacteriia; o__Fusobacteriales; f__Fusobacteriaceae; g__Fusobacterium | 0,03030594 |
| d__Bacteria; p__Bacteroidota; c__Bacteroidia; o__Bacteroidales; f__Prevotellaceae; g__Prevotella; s__Prevotella_pallens | 0,03040758 |
| d__Bacteria; p__Bacteroidota; c__Bacteroidia; o__Bacteroidales; f__Prevotellaceae; g__Prevotella; s__Prevotella_sp._oral_taxon_299_str._F0039 | 0,03046297 |
| d__Bacteria; p__Bacteroidota; c__Bacteroidia; o__Bacteroidales; f__Prevotellaceae; g__Alloprevotella; s__Alloprevotella_tannerae | 0,0307384 |
| d__Bacteria; p__Fusobacteriota; c__Fusobacteriia; o__Fusobacteriales; f__Leptotrichiaceae; g__Leptotrichia | 0,03109264 |
| d__Bacteria; p__Campilobacterota; c__Campylobacteria; o__Campylobacterales; f__Campylobacteraceae; g__Campylobacter; s__uncultured_organism | 0,03110608 |
| d__Bacteria; p__Proteobacteria; c__Gammaproteobacteria; o__Pasteurellales; f__Pasteurellaceae | 0,03133479 |
| d__Bacteria; p__Firmicutes; c__Clostridia; o__Lachnospirales; f__Lachnospiraceae; g__Lachnoanaerobaculum | 0,03150786 |
| d__Bacteria; p__Fusobacteriota; c__Fusobacteriia; o__Fusobacteriales; f__Leptotrichiaceae; g__Leptotrichia | 0,03154387 |
| d__Bacteria; p__Bacteroidota; c__Bacteroidia; o__Bacteroidales; f__Porphyromonadaceae; g__Porphyromonas; s__unidentified | 0,03158753 |
| d__Bacteria; p__Bacteroidota; c__Bacteroidia; o__Bacteroidales; f__Prevotellaceae; g__Alloprevotella | 0,03160641 |
| d__Bacteria; p__Bacteroidota; c__Bacteroidia; o__Bacteroidales; f__Prevotellaceae; g__Alloprevotella | 0,03162344 |
| d__Bacteria; p__Fusobacteriota; c__Fusobacteriia; o__Fusobacteriales; f__Fusobacteriaceae; g__Fusobacterium | 0,03162393 |
| d__Bacteria; p__Bacteroidota; c__Bacteroidia; o__Bacteroidales; f__Porphyromonadaceae; g__Porphyromonas; s__unidentified | 0,03170422 |
| d__Bacteria; p__Firmicutes; c__Bacilli; o__Erysipelotrichales; f__Erysipelotrichaceae; g__Solobacterium; s__Solobacterium_moorei | 0,03192926 |
| d__Bacteria; p__Bacteroidota; c__Bacteroidia; o__Bacteroidales; f__Prevotellaceae; g__Prevotella; s__Prevotella_sp._GEJ23 | 0,03195763 |
| d__Bacteria; p__Patescibacteria; c__Gracilibacteria; o__Absconditabacteriales_(SR1); f__Absconditabacteriales_(SR1); g__Absconditabacteriales_(SR1); s__SR1_bacterium_oral_taxon_875 | 0,03206393 |
| d__Bacteria; p__Bacteroidota; c__Bacteroidia; o__Bacteroidales; f__Paludibacteraceae; g__F0058;Ambiguous_taxa | 0,03213637 |
| d__Bacteria; p__Campilobacterota; c__Campylobacteria; o__Campylobacterales; f__Campylobacteraceae; g__Campylobacter | 0,03221343 |
| d__Bacteria; p__Firmicutes; c__Bacilli; o__Mycoplasmatales; f__Mycoplasmataceae; g__Mycoplasma | 0,03222192 |
| d__Bacteria; p__Firmicutes; c__Negativicutes; o__Veillonellales-Selenomonadales; f__Veillonellaceae; g__Veillonella | 0,03224206 |
| d__Bacteria; p__Campilobacterota; c__Campylobacteria; o__Campylobacterales; f__Campylobacteraceae; g__Campylobacter; s__unidentified | 0,03228583 |
| d__Bacteria; p__Firmicutes; c__Negativicutes; o__Veillonellales-Selenomonadales; f__Veillonellaceae; g__Veillonella | 0,03236928 |
| d__Bacteria; p__Fusobacteriota; c__Fusobacteriia; o__Fusobacteriales; f__Fusobacteriaceae; g__Fusobacterium | 0,03260373 |
| d__Bacteria; p__Fusobacteriota; c__Fusobacteriia; o__Fusobacteriales; f__Fusobacteriaceae; g__Fusobacterium | 0,03262663 |
| d__Bacteria; p__Bacteroidota; c__Bacteroidia; o__Bacteroidales; f__Prevotellaceae; g__Prevotella; s__Prevotella_veroralis | 0,03267974 |
| d__Bacteria; p__Bacteroidota; c__Bacteroidia; o__Bacteroidales; f__Porphyromonadaceae; g__Porphyromonas; s__Porphyromonas_sp._oral_clone_DP023 | 0,03293872 |
| d__Bacteria; p__Fusobacteriota; c__Fusobacteriia; o__Fusobacteriales; f__Fusobacteriaceae; g__Fusobacterium; s__Fusobacterium_sp._Marseille-P2749 | 0,03306878 |
| d__Bacteria; p__Fusobacteriota; c__Fusobacteriia; o__Fusobacteriales; f__Fusobacteriaceae; g__Fusobacterium | 0,03313453 |
| d__Bacteria; p__Bacteroidota; c__Bacteroidia; o__Bacteroidales; f__Prevotellaceae; g__Prevotella; s__Prevotella_veroralis | 0,03322622 |
| d__Bacteria; p__Proteobacteria; c__Gammaproteobacteria; o__Burkholderiales; f__Neisseriaceae; g__Neisseria | 0,03342698 |
| d__Bacteria; p__Bacteroidota; c__Bacteroidia; o__Bacteroidales; f__Porphyromonadaceae; g__Porphyromonas | 0,03389701 |
| d__Bacteria; p__Firmicutes; c__Negativicutes; o__Veillonellales-Selenomonadales; f__Veillonellaceae; g__Veillonella | 0,03393039 |
| d__Bacteria; p__Campilobacterota; c__Campylobacteria; o__Campylobacterales; f__Campylobacteraceae; g__Campylobacter | 0,03398917 |
| d__Bacteria; p__Proteobacteria; c__Gammaproteobacteria; o__Burkholderiales; f__Neisseriaceae; g__Neisseria | 0,03402022 |
| d__Bacteria; p__Firmicutes; c__Negativicutes; o__Veillonellales-Selenomonadales; f__Veillonellaceae; g__Veillonella | 0,03413094 |
| d__Bacteria; p__Bacteroidota; c__Bacteroidia; o__Bacteroidales; f__Porphyromonadaceae; g__Porphyromonas; s__Porphyromonas_gingivalis | 0,03425023 |
| d__Bacteria; p__Bacteroidota; c__Bacteroidia; o__Bacteroidales; f__Prevotellaceae; g__Prevotella; s__Prevotella_sp._GEJ23 | 0,03453039 |
| d__Bacteria; p__Bacteroidota; c__Bacteroidia; o__Bacteroidales; f__Prevotellaceae; g__Prevotella; s__Prevotella_sp._oral_taxon_299_str._F0039 | 0,03456547 |
| d__Bacteria; p__Bacteroidota; c__Bacteroidia; o__Bacteroidales; f__Prevotellaceae; g__Prevotella | 0,03474434 |
| d__Bacteria; p__Proteobacteria; c__Gammaproteobacteria; o__Burkholderiales; f__Neisseriaceae; g__Neisseria | 0,03485778 |
| d__Bacteria; p__Campilobacterota; c__Campylobacteria; o__Campylobacterales; f__Campylobacteraceae; g__Campylobacter; s__uncultured_organism | 0,03507833 |
| d__Bacteria; p__Proteobacteria; c__Gammaproteobacteria; o__Pasteurellales; f__Pasteurellaceae | 0,03517269 |
| d__Bacteria; p__Bacteroidota; c__Bacteroidia; o__Bacteroidales; f__Prevotellaceae; g__Prevotella; s__Prevotella_sp._oral_taxon_299_str._F0039 | 0,03520543 |
| d__Bacteria; p__Firmicutes; c__Negativicutes; o__Veillonellales-Selenomonadales; f__Veillonellaceae; g__Veillonella | 0,03525023 |
| d__Bacteria; p__Bacteroidota; c__Bacteroidia; o__Bacteroidales; f__Prevotellaceae; g__Alloprevotella | 0,03530095 |
| d__Bacteria; p__Fusobacteriota; c__Fusobacteriia; o__Fusobacteriales; f__Fusobacteriaceae; g__Fusobacterium | 0,03535147 |
| d__Bacteria; p__Bacteroidota; c__Bacteroidia; o__Bacteroidales; f__Prevotellaceae; g__Prevotella; s__Prevotella_sp._oral_taxon_299_str._F0039 | 0,0354188 |
| d__Bacteria; p__Firmicutes; c__Negativicutes; o__Veillonellales-Selenomonadales; f__Veillonellaceae; g__Megasphaera; s__Megasphaera_micronuciformis | 0,03562034 |
| d__Bacteria; p__Firmicutes; c__Bacilli; o__Lactobacillales; f__Streptococcaceae; g__Streptococcus | 0,0357296 |
| d__Bacteria; p__Bacteroidota; c__Bacteroidia; o__Bacteroidales; f__Prevotellaceae; g__Alloprevotella; s__Prevotella_sp. | 0,03574713 |
| d__Bacteria; p__Proteobacteria; c__Gammaproteobacteria; o__Pasteurellales; f__Pasteurellaceae; g__Actinobacillus | 0,03575807 |
| d__Bacteria; p__Proteobacteria; c__Gammaproteobacteria; o__Pasteurellales; f__Pasteurellaceae; g__Actinobacillus | 0,03575807 |
| d__Bacteria; p__Proteobacteria; c__Gammaproteobacteria; o__Burkholderiales; f__Neisseriaceae; g__Neisseria | 0,03590205 |
| d__Bacteria; p__Firmicutes; c__Clostridia; o__Peptostreptococcales-Tissierellales; f__Peptostreptococcaceae; g__Peptostreptococcus | 0,03604943 |
| d__Bacteria; p__Bacteroidota; c__Bacteroidia; o__Bacteroidales; f__Porphyromonadaceae; g__Porphyromonas; s__Porphyromonas_sp._oral_clone_DP023 | 0,03605803 |
| d__Bacteria; p__Campilobacterota; c__Campylobacteria; o__Campylobacterales; f__Campylobacteraceae; g__Campylobacter; s__unidentified | 0,03619019 |
| d__Bacteria; p__Bacteroidota; c__Bacteroidia; o__Bacteroidales; f__Porphyromonadaceae; g__Porphyromonas; s__Porphyromonas_sp._oral_clone_DP023 | 0,03624017 |
| d__Bacteria; p__Bacteroidota; c__Bacteroidia; o__Bacteroidales; f__Prevotellaceae; g__Alloprevotella; s__Prevotella_sp._oral_clone_BU035 | 0,03644122 |
| d__Bacteria; p__Proteobacteria; c__Gammaproteobacteria; o__Burkholderiales; f__Neisseriaceae | 0,03649525 |
| d__Bacteria; p__Firmicutes; c__Clostridia; o__Lachnospirales; f__Lachnospiraceae; g__Stomatobaculum | 0,0366426 |
| d__Bacteria; p__Bacteroidota; c__Bacteroidia; o__Bacteroidales; f__Prevotellaceae; g__Alloprevotella | 0,03667755 |
| d__Bacteria; p__Fusobacteriota; c__Fusobacteriia; o__Fusobacteriales; f__Leptotrichiaceae; g__Leptotrichia; s__Leptotrichia_sp._oral_clone_FP036 | 0,03669053 |
| d__Bacteria; p__Firmicutes; c__Negativicutes; o__Veillonellales-Selenomonadales; f__Veillonellaceae; g__Veillonella | 0,03677652 |
| d__Bacteria; p__Proteobacteria; c__Gammaproteobacteria; o__Pasteurellales; f__Pasteurellaceae; g__Haemophilus | 0,03717267 |
| d__Bacteria; p__Firmicutes; c__Negativicutes; o__Veillonellales-Selenomonadales; f__Veillonellaceae; g__Veillonella | 0,03718017 |
| d__Bacteria; p__Fusobacteriota; c__Fusobacteriia; o__Fusobacteriales; f__Fusobacteriaceae; g__Fusobacterium | 0,03718682 |
| d__Bacteria; p__Bacteroidota; c__Bacteroidia; o__Bacteroidales; f__Prevotellaceae; g__Prevotella; s__Prevotella_pallens | 0,03748437 |
| d__Bacteria; p__Bacteroidota; c__Bacteroidia; o__Bacteroidales; f__Prevotellaceae; g__Prevotella; s__Prevotella_sp._oral_taxon_299_str._F0039 | 0,03793645 |
| d__Bacteria; p__Bacteroidota; c__Bacteroidia; o__Bacteroidales; f__Prevotellaceae; g__Alloprevotella; s__Prevotella_sp. | 0,03828275 |
| d__Bacteria; p__Firmicutes; c__Bacilli; o__Lactobacillales; f__Streptococcaceae; g__Streptococcus | 0,03851577 |
| d__Bacteria; p__Bacteroidota; c__Bacteroidia; o__Bacteroidales; f__Prevotellaceae; g__Prevotella; s__Prevotella_sp._oral_taxon_299_str._F0039 | 0,03866618 |
| d__Bacteria; p__Proteobacteria; c__Gammaproteobacteria; o__Burkholderiales; f__Neisseriaceae; g__Neisseria | 0,0387807 |
| d__Bacteria; p__Firmicutes; c__Negativicutes; o__Veillonellales-Selenomonadales; f__Veillonellaceae; g__Veillonella | 0,03879835 |
| d__Bacteria; p__Bacteroidota; c__Bacteroidia; o__Bacteroidales; f__Porphyromonadaceae; g__Porphyromonas; s__Porphyromonas_sp._oral_clone_DP023 | 0,03894593 |
| d__Bacteria; p__Firmicutes; c__Negativicutes; o__Veillonellales-Selenomonadales; f__Veillonellaceae; g__Megasphaera; s__Megasphaera_micronuciformis | 0,03932313 |
| d__Bacteria; p__Bacteroidota; c__Bacteroidia; o__Bacteroidales; f__Prevotellaceae; g__Alloprevotella; s__Prevotella_sp. | 0,03945707 |
| d__Bacteria; p__Fusobacteriota; c__Fusobacteriia; o__Fusobacteriales; f__Fusobacteriaceae; g__Fusobacterium | 0,04028449 |
| d__Bacteria; p__Fusobacteriota; c__Fusobacteriia; o__Fusobacteriales; f__Fusobacteriaceae; g__Fusobacterium | 0,04049081 |
| d__Bacteria; p__Proteobacteria; c__Gammaproteobacteria; o__Pasteurellales; f__Pasteurellaceae; g__Actinobacillus | 0,04086637 |
| d__Bacteria; p__Proteobacteria; c__Gammaproteobacteria; o__Burkholderiales; f__Neisseriaceae; g__Neisseria | 0,04131026 |
| d__Bacteria; p__Firmicutes; c__Bacilli; o__Lactobacillales; f__Streptococcaceae; g__Streptococcus | 0,04167411 |
| d__Bacteria; p__Firmicutes; c__Clostridia; o__Peptostreptococcales-Tissierellales; f__Peptostreptococcaceae; g__Filifactor;Ambiguous_taxa | 0,04171339 |
| d__Bacteria; p__Bacteroidota; c__Bacteroidia; o__Bacteroidales; f__Prevotellaceae; g__Prevotella; s__Prevotella_sp._oral_taxon_299_str._F0039 | 0,0418046 |
| d__Bacteria; p__Firmicutes; c__Bacilli; o__Lactobacillales; f__Streptococcaceae; g__Streptococcus | 0,04187209 |
| d__Bacteria; p__Patescibacteria; c__Gracilibacteria; o__Absconditabacteriales_(SR1); f__Absconditabacteriales_(SR1); g__Absconditabacteriales_(SR1); s__SR1_bacterium_oral_taxon_875 | 0,04192976 |
| d__Bacteria; p__Firmicutes; c__Negativicutes; o__Veillonellales-Selenomonadales; f__Veillonellaceae; g__Megasphaera; s__Megasphaera_micronuciformis | 0,04225621 |
| d__Bacteria; p__Firmicutes; c__Negativicutes; o__Veillonellales-Selenomonadales; f__Veillonellaceae; g__Megasphaera; s__Megasphaera_micronuciformis | 0,04230329 |
| d__Bacteria; p__Firmicutes; c__Bacilli; o__Lactobacillales; f__Streptococcaceae | 0,0428791 |
| d__Bacteria; p__Bacteroidota; c__Bacteroidia; o__Bacteroidales; f__Prevotellaceae; g__Prevotella | 0,04335862 |
| d__Bacteria; p__Bacteroidota; c__Bacteroidia; o__Bacteroidales; f__Porphyromonadaceae; g__Porphyromonas; s__Porphyromonas_sp._oral_clone_DP023 | 0,04368534 |
| d__Bacteria; p__Bacteroidota; c__Bacteroidia; o__Bacteroidales; f__Prevotellaceae; g__Alloprevotella; s__Prevotella_sp. | 0,04375866 |
| d__Bacteria; p__Bacteroidota; c__Bacteroidia; o__Bacteroidales; f__Prevotellaceae; g__Prevotella; s__Prevotella_pallens | 0,04385897 |
| d__Bacteria; p__Bacteroidota; c__Bacteroidia; o__Bacteroidales; f__Prevotellaceae; g__Prevotella; s__Prevotella_sp._GEJ23 | 0,044475 |
| d__Bacteria; p__Firmicutes; c__Negativicutes; o__Veillonellales-Selenomonadales; f__Veillonellaceae; g__Veillonella | 0,04464286 |
| d__Bacteria; p__Firmicutes; c__Clostridia; o__Lachnospirales; f__Lachnospiraceae; g__Lachnoanaerobaculum; s__uncultured_organism | 0,04466035 |
| d__Bacteria; p__Firmicutes; c__Clostridia; o__Peptostreptococcales-Tissierellales; f__Peptostreptococcales-Tissierellales; g__Parvimonas | 0,04482621 |
| d__Bacteria; p__Bacteroidota; c__Bacteroidia; o__Bacteroidales; f__Porphyromonadaceae; g__Porphyromonas; s__unidentified | 0,04523411 |
| d__Bacteria; p__Bacteroidota; c__Bacteroidia; o__Bacteroidales; f__Prevotellaceae; g__Alloprevotella; s__Prevotella_sp. | 0,04531511 |
| d__Bacteria; p__Bacteroidota; c__Bacteroidia; o__Bacteroidales; f__Prevotellaceae; g__Prevotella; s__Prevotella_sp._oral_taxon_299_str._F0039 | 0,0457264 |
| d__Bacteria; p__Bacteroidota; c__Bacteroidia; o__Bacteroidales; f__Prevotellaceae; g__Alloprevotella | 0,04575555 |
| d__Bacteria; p__Proteobacteria; c__Gammaproteobacteria; o__Pasteurellales; f__Pasteurellaceae | 0,04585674 |
| d__Bacteria; p__Proteobacteria; c__Gammaproteobacteria; o__Pasteurellales; f__Pasteurellaceae | 0,04603591 |
| d__Bacteria; p__Bacteroidota; c__Bacteroidia; o__Bacteroidales; f__Prevotellaceae; g__Prevotella; s__Prevotella_pallens | 0,0462963 |
| d__Bacteria; p__Bacteroidota; c__Bacteroidia; o__Bacteroidales; f__Prevotellaceae; g__Prevotella; s__Prevotella_pallens | 0,04690699 |
| d__Bacteria; p__Firmicutes; c__Negativicutes; o__Veillonellales-Selenomonadales; f__Veillonellaceae; g__Veillonella | 0,04749845 |
| d__Bacteria; p__Firmicutes; c__Bacilli; o__Lactobacillales; f__Streptococcaceae | 0,04808585 |
| d__Bacteria; p__Fusobacteriota; c__Fusobacteriia; o__Fusobacteriales; f__Fusobacteriaceae; g__Fusobacterium | 0,04824212 |
| d__Bacteria; p__Bacteroidota; c__Bacteroidia; o__Bacteroidales; f__Prevotellaceae; g__Alloprevotella | 0,04870998 |
| d__Bacteria; p__Bacteroidota; c__Bacteroidia; o__Bacteroidales; f__Prevotellaceae; g__Prevotella; s__Prevotella_intermedia | 0,04887762 |
| d__Bacteria; p__Fusobacteriota; c__Fusobacteriia; o__Fusobacteriales; f__Fusobacteriaceae; g__Fusobacterium | 0,04919849 |
| d__Bacteria; p__Patescibacteria; c__Gracilibacteria; o__Absconditabacteriales_(SR1); f__Absconditabacteriales_(SR1); g__Absconditabacteriales_(SR1); s__SR1_bacterium_oral_taxon_875 | 0,04932912 |
| d__Bacteria; p__Bacteroidota; c__Bacteroidia; o__Bacteroidales; f__Prevotellaceae; g__Alloprevotella; s__Prevotella_sp. | 0,04995264 |
| d__Bacteria; p__Firmicutes; c__Bacilli; o__Erysipelotrichales; f__Erysipelotrichaceae; g__Solobacterium; s__Solobacterium_moorei | 0,05012326 |
| d__Bacteria; p__Firmicutes; c__Negativicutes; o__Veillonellales-Selenomonadales; f__Veillonellaceae; g__Veillonella | 0,05065513 |
| d__Bacteria; p__Bacteroidota; c__Bacteroidia; o__Bacteroidales; f__Prevotellaceae; g__Prevotella; s__Prevotella_sp._oral_taxon_299_str._F0039 | 0,05066123 |
| d__Bacteria; p__Bacteroidota; c__Bacteroidia; o__Bacteroidales; f__Prevotellaceae; g__Prevotella; s__Prevotella_intermedia | 0,05112946 |
| d__Bacteria; p__Bacteroidota; c__Bacteroidia; o__Bacteroidales; f__Prevotellaceae; g__Prevotella; s__Prevotella_intermedia | 0,05128314 |
| d__Bacteria; p__Bacteroidota; c__Bacteroidia; o__Bacteroidales; f__Prevotellaceae; g__Prevotella; s__Prevotella_pallens | 0,05163563 |
| d__Bacteria; p__Fusobacteriota; c__Fusobacteriia; o__Fusobacteriales; f__Leptotrichiaceae; g__Leptotrichia | 0,05200886 |
| d__Bacteria; p__Bacteroidota; c__Bacteroidia; o__Bacteroidales; f__Porphyromonadaceae; g__Porphyromonas; s__Porphyromonas_sp._oral_clone_DP023 | 0,05279497 |
| d__Bacteria; p__Bacteroidota; c__Bacteroidia; o__Bacteroidales; f__Porphyromonadaceae; g__Porphyromonas; s__Porphyromonas_sp._oral_clone_DP023 | 0,05299531 |
| d__Bacteria; p__Bacteroidota; c__Bacteroidia; o__Bacteroidales; f__Prevotellaceae; g__Alloprevotella; s__Prevotella_sp._oral_clone_BU035 | 0,05419486 |
| d__Bacteria; p__Bacteroidota; c__Bacteroidia; o__Bacteroidales; f__Prevotellaceae; g__Prevotella; s__Prevotella_sp._oral_taxon_299_str._F0039 | 0,05455564 |
| d__Bacteria; p__Firmicutes; c__Bacilli; o__Lactobacillales; f__Streptococcaceae; g__Streptococcus | 0,05545973 |
| d__Bacteria; p__Proteobacteria; c__Gammaproteobacteria; o__Pasteurellales; f__Pasteurellaceae; g__Actinobacillus | 0,05619125 |
| d__Bacteria; p__Firmicutes; c__Bacilli; o__Lactobacillales; f__Streptococcaceae; g__Streptococcus | 0,05653501 |
| d__Bacteria; p__Bacteroidota; c__Bacteroidia; o__Bacteroidales; f__Prevotellaceae; g__Prevotella; s__Prevotella_sp._oral_taxon_299_str._F0039 | 0,05721818 |
| d__Bacteria; p__Firmicutes; c__Negativicutes; o__Veillonellales-Selenomonadales; f__Veillonellaceae; g__Veillonella | 0,05722814 |
| d__Bacteria; p__Firmicutes; c__Clostridia; o__Peptostreptococcales-Tissierellales; f__Peptostreptococcaceae; g__Peptostreptococcus | 0,0576293 |
| d__Bacteria; p__Firmicutes; c__Clostridia; o__Peptostreptococcales-Tissierellales; f__Peptostreptococcaceae; g__Peptostreptococcus | 0,05810097 |
| d__Bacteria; p__Bacteroidota; c__Bacteroidia; o__Bacteroidales; f__Prevotellaceae; g__Prevotella; s__Prevotella_sp._oral_taxon_299_str._F0039 | 0,05814594 |
| d__Bacteria; p__Actinobacteriota; c__Actinobacteria; o__Micrococcales; f__Micrococcaceae; g__Rothia | 0,0585921 |
| d__Bacteria; p__Bacteroidota; c__Bacteroidia; o__Bacteroidales; f__Prevotellaceae; g__Prevotella; s__Prevotella_sp._oral_taxon_299_str._F0039 | 0,05859563 |
| d__Bacteria; p__Bacteroidota; c__Bacteroidia; o__Bacteroidales; f__Prevotellaceae; g__Prevotella; s__Prevotella_sp._GEJ23 | 0,05883254 |
| d__Bacteria; p__Bacteroidota; c__Bacteroidia; o__Bacteroidales; f__Prevotellaceae; g__Prevotella; s__Prevotella_sp._GEJ23 | 0,05966556 |
| d__Bacteria; p__Bacteroidota; c__Bacteroidia; o__Bacteroidales; f__Prevotellaceae; g__Alloprevotella; s__Prevotella_sp. | 0,06202545 |
| d__Bacteria; p__Bacteroidota; c__Bacteroidia; o__Bacteroidales; f__Prevotellaceae; g__Alloprevotella | 0,06217222 |
| d__Bacteria; p__Firmicutes; c__Bacilli; o__Lactobacillales; f__Streptococcaceae; g__Streptococcus | 0,06401902 |
| d__Bacteria; p__Patescibacteria; c__Gracilibacteria; o__Absconditabacteriales_(SR1); f__Absconditabacteriales_(SR1); g__Absconditabacteriales_(SR1); s__SR1_bacterium_oral_taxon_875 | 0,06412786 |
| d__Bacteria; p__Bacteroidota; c__Bacteroidia; o__Bacteroidales; f__Prevotellaceae; g__Prevotella; s__Prevotella_pallens | 0,06432602 |
| d__Bacteria; p__Bacteroidota; c__Bacteroidia; o__Bacteroidales; f__Prevotellaceae; g__Prevotella; s__Prevotella_veroralis | 0,06570057 |
| d__Bacteria; p__Patescibacteria; c__Gracilibacteria; o__Absconditabacteriales_(SR1); f__Absconditabacteriales_(SR1); g__Absconditabacteriales_(SR1); s__SR1_bacterium_oral_taxon_875 | 0,06659432 |
| d__Bacteria; p__Firmicutes; c__Bacilli; o__Lactobacillales; f__Streptococcaceae; g__Streptococcus | 0,06673129 |
| d__Bacteria; p__Bacteroidota; c__Bacteroidia; o__Bacteroidales; f__Prevotellaceae; g__Prevotella; s__Prevotella_sp._oral_taxon_299_str._F0039 | 0,06699718 |
| d__Bacteria; p__Firmicutes; c__Bacilli; o__Lactobacillales; f__Streptococcaceae | 0,06731075 |
| d__Bacteria; p__Firmicutes; c__Negativicutes; o__Veillonellales-Selenomonadales; f__Veillonellaceae; g__Veillonella | 0,07066055 |
| d__Bacteria; p__Proteobacteria; c__Gammaproteobacteria; o__Burkholderiales; f__Neisseriaceae; g__Neisseria | 0,07078695 |
| d__Bacteria; p__Firmicutes; c__Bacilli; o__Staphylococcales; f__Gemellaceae; g__Gemella | 0,0720826 |
| d__Bacteria; p__Fusobacteriota; c__Fusobacteriia; o__Fusobacteriales; f__Fusobacteriaceae; g__Fusobacterium | 0,07535288 |
| d__Bacteria; p__Bacteroidota; c__Bacteroidia; o__Bacteroidales; f__Prevotellaceae; g__Alloprevotella; s__Alloprevotella_tannerae | 0,07592131 |
| d__Bacteria; p__Proteobacteria; c__Gammaproteobacteria; o__Pasteurellales; f__Pasteurellaceae | 0,07700344 |
| d__Bacteria; p__Bacteroidota; c__Bacteroidia; o__Bacteroidales; f__Prevotellaceae; g__Alloprevotella; s__Prevotella_sp. | 0,0782809 |
| d__Bacteria; p__Bacteroidota; c__Bacteroidia; o__Bacteroidales; f__Prevotellaceae; g__Prevotella; s__Prevotella_pallens | 0,07908949 |
| d__Bacteria; p__Firmicutes; c__Negativicutes; o__Veillonellales-Selenomonadales; f__Veillonellaceae; g__Veillonella | 0,08031081 |
| d__Bacteria; p__Bacteroidota; c__Bacteroidia; o__Bacteroidales; f__Prevotellaceae; g__Alloprevotella; s__Prevotella_sp. | 0,08122962 |
| d__Bacteria; p__Bacteroidota; c__Bacteroidia; o__Bacteroidales; f__Porphyromonadaceae; g__Porphyromonas; s__unidentified | 0,08210847 |
| d__Bacteria; p__Firmicutes; c__Negativicutes; o__Veillonellales-Selenomonadales; f__Veillonellaceae; g__Veillonella | 0,08220818 |
| d__Bacteria; p__Firmicutes; c__Negativicutes; o__Veillonellales-Selenomonadales; f__Veillonellaceae; g__Veillonella | 0,08246181 |
| d__Bacteria; p__Bacteroidota; c__Bacteroidia; o__Bacteroidales; f__Prevotellaceae; g__Prevotella; s__Prevotella_intermedia | 0,08459163 |
| d__Bacteria; p__Proteobacteria; c__Gammaproteobacteria; o__Pasteurellales; f__Pasteurellaceae | 0,08540416 |
| d__Bacteria; p__Bacteroidota; c__Bacteroidia; o__Bacteroidales; f__Prevotellaceae; g__Alloprevotella | 0,08670935 |
| d__Bacteria; p__Firmicutes; c__Bacilli; o__Lactobacillales; f__Streptococcaceae; g__Streptococcus | 0,08798677 |
| d__Bacteria; p__Proteobacteria; c__Gammaproteobacteria; o__Burkholderiales; f__Neisseriaceae; g__Neisseria; s__Neisseria_flavescens | 0,08991058 |
| d__Bacteria; p__Bacteroidota; c__Bacteroidia; o__Bacteroidales; f__Prevotellaceae; g__Prevotella; s__Prevotella_sp._oral_taxon_299_str._F0039 | 0,09202429 |
| d__Bacteria; p__Proteobacteria; c__Gammaproteobacteria; o__Burkholderiales; f__Neisseriaceae; g__Neisseria | 0,10102208 |
| d__Bacteria; p__Proteobacteria; c__Gammaproteobacteria; o__Burkholderiales; f__Neisseriaceae; g__Neisseria | 0,10380038 |
| d__Bacteria; p__Fusobacteriota; c__Fusobacteriia; o__Fusobacteriales; f__Fusobacteriaceae; g__Fusobacterium | 0,1076125 |
| d__Bacteria; p__Firmicutes; c__Negativicutes; o__Veillonellales-Selenomonadales; f__Veillonellaceae; g__Veillonella | 0,11269848 |
| d__Bacteria; p__Bacteroidota; c__Bacteroidia; o__Bacteroidales; f__Prevotellaceae; g__Prevotella; s__Prevotella_sp._oral_taxon_299_str._F0039 | 0,11381797 |
| d__Bacteria; p__Proteobacteria; c__Gammaproteobacteria; o__Burkholderiales; f__Neisseriaceae; g__Neisseria | 0,11708333 |
| d__Bacteria; p__Proteobacteria; c__Gammaproteobacteria; o__Pasteurellales; f__Pasteurellaceae; g__Actinobacillus | 0,11749081 |
| d__Bacteria; p__Firmicutes; c__Negativicutes; o__Veillonellales-Selenomonadales; f__Veillonellaceae; g__Veillonella | 0,12165745 |
| d__Bacteria; p__Bacteroidota; c__Bacteroidia; o__Bacteroidales; f__Prevotellaceae; g__Prevotella; s__Prevotella_veroralis | 0,14272838 |
| d__Bacteria; p__Bacteroidota; c__Bacteroidia; o__Bacteroidales; f__Porphyromonadaceae; g__Porphyromonas; s__Porphyromonas_sp._oral_clone_DP023 | 0,15123525 |
| d__Bacteria; p__Firmicutes; c__Negativicutes; o__Veillonellales-Selenomonadales; f__Veillonellaceae; g__Veillonella | 0,17451165 |
| d__Bacteria; p__Proteobacteria; c__Gammaproteobacteria; o__Burkholderiales; f__Neisseriaceae; g__Neisseria | 0,17724353 |

| **Table S3 and attached box plot**. Sequencing data from viral and microbial metagenomes (raw sequencing data). Quality read data is shown below in the attached box plot (global ditributions of reads lengths and qualities among all samples) | | | |
| --- | --- | --- | --- |
| Sample group | Total sequenced nucleotides (Mb) | No. of raw reads | Mean read length (bp) |
| viral metagenomes (Controls) | 6704 | 47144866 | 142.2 |
| viral metagenomes (IgA deficiency) | 5900 | 41696113 | 141.5 |
| Microbial metagenomes (Controls) | 44672 | 310715490 | 143.77 |
| Microbial metagenomes (IgA deficiency) | 50164 | 353845192 | 141.76 |


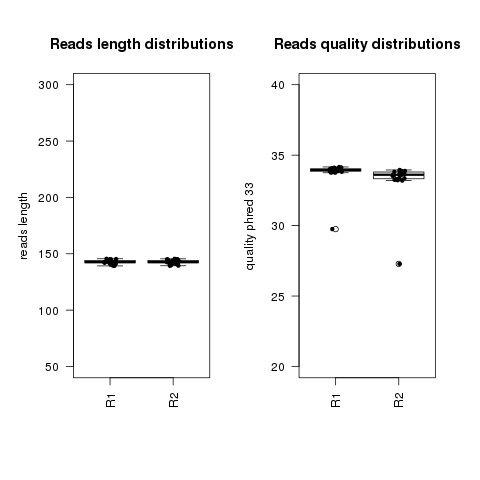

Supplement: Supplementary file 1 — Supplementary Information. [file 41598_2021_94507_MOESM1_ESM.docx]
